# Valley-spin polarization at zero magnetic field induced by strong hole-hole interactions in monolayer WSe_2_

**DOI:** 10.1126/sciadv.adu4696

**Published:** 2025-05-07

**Authors:** Justin Boddison-Chouinard, Marek Korkusinski, Alex Bogan, Pedro Barrios, Philip Waldron, Kenji Watanabe, Takashi Taniguchi, Jarosław Pawłowski, Daniel Miravet, Pawel Hawrylak, Adina Luican-Mayer, Louis Gaudreau

**Affiliations:** ^1^ Quantum and Nanotechnologies Research Centre, National Research Council Canada, Ottawa, Ontario, K1A 0R6, Canada.; ^2^ Department of Physics, University of Ottawa, Ottawa, Ontario, K1N 9A7, Canada.; ^3^ Research Center for Electronic and Optical Materials, National Institute for Materials Science, 1-1 Namiki, Tsukuba 305-0044, Japan.; ^4^ Research Center for Materials Nanoarchitectonics, National Institute for Materials Science, 1-1 Namiki, Tsukuba 305-0044, Japan.; ^5^ Institute of Theoretical Physics, Wrocław University of Science and Technology, Wrocław, Poland.; ^6^ Materials Science Division, Argonne National Laboratory, Lemont, IL 60439, USA.

## Abstract

Monolayer transition metal dichalcogenides have emerged as prominent candidates to explore the complex interplay between spin and valley degrees of freedom. Their strong spin-orbit interaction and broken inversion symmetry lead to the spin-valley locking effect, in which carriers occupying the *K* and *K*′ valleys of the reciprocal space must have opposite spins. This effect is particularly strong for holes due to a larger spin-orbit gap in the valence band. By reducing the dimensionality of a monolayer of WSe_2_ to 1D via electrostatic confinement, we demonstrate that spin-valley locking and strong hole-hole interactions lead to a ferromagnetic state where hole transport is spin-valley polarized, even without an applied magnetic field. A massive Dirac fermion model in the Hartree-Fock approximation reveals that many-body hole-exchange interactions lead to this polarized ground-state. This observation opens the possibility of implementing a robust and stable valley-polarized system, essential in valleytronic applications.

## Teaser

Strong hole-hole interactions and spin-orbit coupling lead to a valley-polarized state in 1D confined monolayer WSe_2_.

## INTRODUCTION

Charge, spin, and valley degrees of freedom are fundamental in condensed matter physics not only because they enable storing and processing information in devices but also because their interplay can lead to exotic phases of matter. Despite advancements, there are still challenges in understanding and manipulating these interactions, particularly in the context of quantum technologies and quantum confined device architectures, where they can be affected by quantization effects, modified coupling strengths or many body interactions. Among material platforms, two-dimensional (2D) transition metal dichalcogenides (TMDs) emerged as particularly attractive for spintronics and valleytronics (*1*–*9*). One of the most remarkable properties of monolayer and odd multilayer TMDs is that due to strong spin-orbit interaction and broken spatial inversion symmetry, the *K* and *K*′ valleys of the Brillouin zone are spin-split at zero magnetic field (*10*). This splitting is particularly strong for holes in the valence band reaching ≈450 meV (*11*, *12*), which leads to robust spin-valley locking where spin-up (↑) holes reside in the *K* valley and spin-down (↓) holes in the *K*′ valley. While early observations of spin-valley locking were achieved in 2D devices using optical probes (*13*–*15*) and transport (*16*, *17*), only more recent progress in device fabrication permitted addressing this phenomenon in quantum confined architectures (*18*–*23*); however, many of the observed features remain elusive.

Here, we combine experimental evidence with theoretical calculations and demonstrate the role that spin-valley locking plays in the properties of 1D transport in monolayer tungsten diselenide (WSe_2_). We show how hole-hole interactions lead to spin-valley–polarized transport at zero magnetic field that can be tuned by controlling the carrier density in the device. We show that in TMDs, the combination of spin-valley locking and strong exchange interactions are two ingredients that are sufficient to fully understand spin-valley polarization at zero magnetic field.

## RESULTS

### Device architecture and electrical characterization

**Fig. 1.**
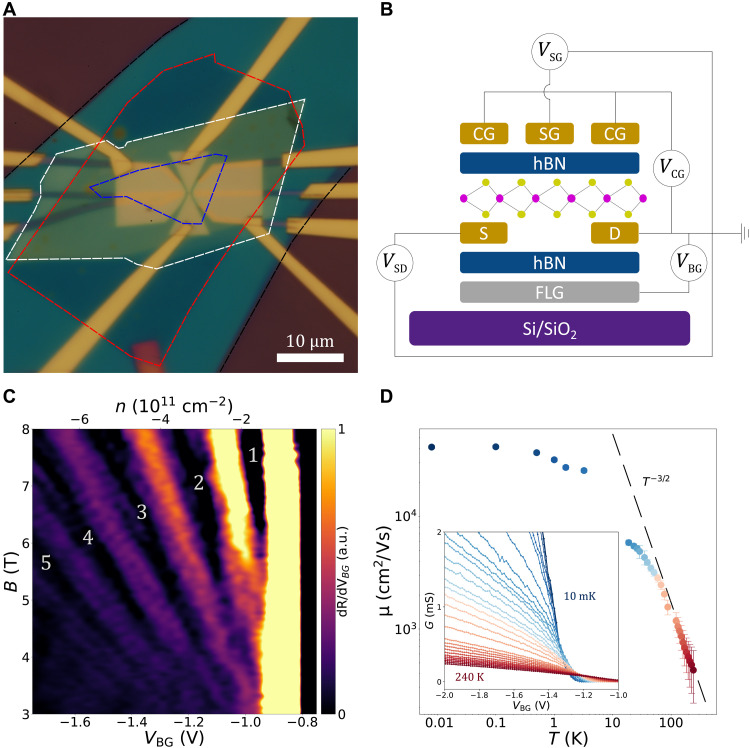
Device architecture and electrical characterization. (**A**) Optical micrograph of the WSe_2_ device where the graphite (red), bottom hBN (black), monolayer WSe_2_ (blue), and top hBN (white) are outlined. (**B**) Schematic illustration of the device architecture. (**C**) Landau fan diagram of the differential resistance $dR/dV_{BG}$ (a.u., arbitrary units) measured at 10 mK. The first five Landau levels are labeled with their respective filling factors. (**D**) Field-effect mobility as a function of temperature extracted from the activation curves plotted in the inset. A dashed line represents $\mu \propto T^{-3/2}$ as a guide, indicating that optical phonons are the dominating scattering mechanism at high temperatures (*27*). The inset shows four-probe conductance measurement as a function of the back-gate voltage when *V*_*SG*_ = 0 V and *V*_*CG*_ = −7 V.

In our experiments, we use a device architecture having a monolayer WSe_2_ encapsulated by hexagonal boron nitride (hBN), electrical contacts prepatterned using chromium/platinum (Cr/Pt), a graphite back-gate, and accumulation and depletion top gates (*23*). Figure 1A shows an optical micrograph of the device, and its cross-sectional view is represented in Fig. 1B, where the top gates can be grouped into contact gates and split gates, labeled as CG and SG. The device contacts are activated by applying a negative voltage to the contact gates which locally increase the hole concentration in the contact region of the WSe_2_. Applying a positive voltage on the split gates depletes the underlying region from holes and defines a tunable 1D transport channel in the monolayer WSe_2_ sheet with a lithographic width of 200 nm and a length of 600 nm. The graphite back-gate tunes the carrier density in the entirety of the WSe_2_ flake with the exception of the areas above the metal contacts due to screening. This results in total tunability of the carrier density without affecting the quality of the contacts. In this study, the contact gates were fixed to −7 V resulting in an average resistance per contact of approximately 2 kilohms at 10 mK (see the Supplementary Materials for contact resistance details). Independent control of the carrier density of the contacts and of the active region enables the study of important transport properties in monolayer WSe_2_, including quantum transport across a 1D channel.

To demonstrate the excellent electronic properties of the device, we first perform Landau level spectroscopy experiments. Figure 1C shows the differential four-point resistance ${dR/dV}_{BG}$ as a function of back-gate voltage (*V*_*BG*_) and perpendicular magnetic field (*B*_⊥_) at 10 mK. We observe Landau quantization at fields as low as 3 T, with the zeroth Landau level (ν = 1, labeled as 1 in Fig. 1C) emerging around 6 T. Low-filling factor integer quantum Hall states have been previously observed in monolayer TMDs using contactless methods such as electronic compressibility measurements (*24*, *25*) but have remained elusive in transport measurements until very recently (*16*, *26*), due to high contact resistances and low mobilities.

Further confirming the high quality of our device, Fig. 1D shows the field-effect mobilities as a function of temperature, which have been calculated from activation curves (inset in Fig. 1D) of our sample using the gate capacitance extracted from the Landau fan diagram in Fig. 1C. At 240 K, we extract a field-effect mobility μ_FE_ = 400 ± 200 cm^2^/(Vs). As the temperature decreases, the mobility increases following the expected power law, μ ∝ *T*^−γ^, with γ ≈ 3/2, indicating that, at higher temperatures, optical phonons are the dominant scattering mechanism (*27*, *28*). For lower temperatures, the mobility saturates, and it is dominated by scattering impurities. At 10 mK, we achieve a mobility of 41,000 ± 2000 cm^2^/(Vs), comparable to recent reports of high-quality flux-grown monolayer WSe_2_ samples (*26*, *29*).

### Magneto-transport properties of a gate-defined 1D channel in monolayer WSe_2_

**Fig. 2.**
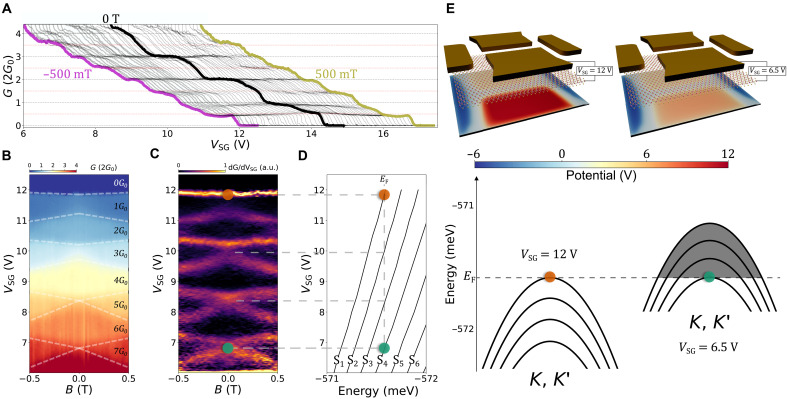
Magneto-transport properties of a gate-defined 1D channel in monolayer WSe_2_. (**A**) Conductance across the 1D channel as a function of the voltage applied to the upper split gate and magnetic field. The magnetic field ranges from −500 mT (purple curve) to 500 mT (gold curve) with a 20-mT interval. The central black curve corresponds to *B*_⊥_ = 0 T. The lower split gate, back-gate, and contact gates are all kept constant at 4, −3.5, and −7 V, respectively. Individual curves are offset by 100 mV along the horizontal axis for clarity. (**B**) Color map of the conductance as a function of the upper split gate voltage and magnetic field. White dashed lines separate regions of different conductance. (**C**) Transconductance as a function of the upper split gate voltage and magnetic field obtained from the derivative of the data in (B). (**D**) Subband edge energies as a function of the upper split gate voltage computed with the anisotropic massive Dirac Fermion model. A black vertical dashed line shows the position of the Fermi energy. (**E**) The potential landscape (top) and resulting subband dispersions (bottom) when *V*_*SG*_ = 12 V and *V*_*SG*_ = 6.5 V. The position of the Fermi energy is indicated by the horizontal dashed line.

**Fig. 3.**
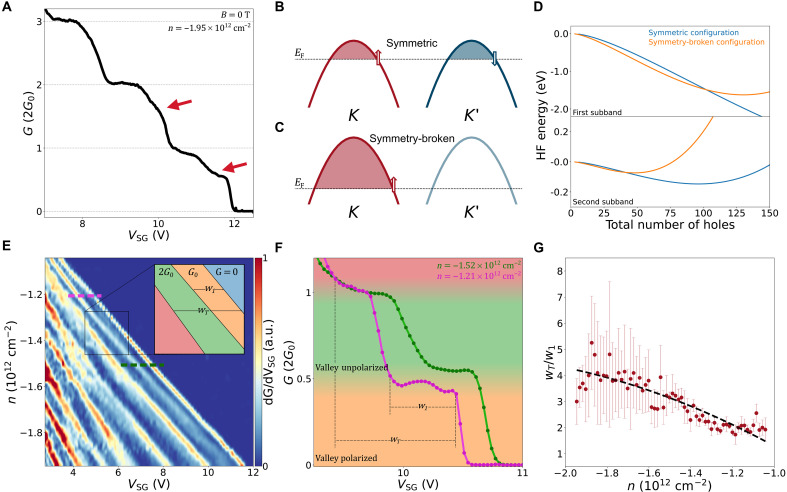
Valley-polarized transport at zero magnetic field. (**A**) Conduction as a function of *V*_*SG*_ at *B*_⊥_ = 0 T where red arrows identify features at *e*^2^/*h* and 3 *e*^2^/*h*. (**B** and **C**) Depictions of two possible configurations for which holes can enter the system. In (B), the symmetric configuration, both valleys are populated, and each contribute *G*_0_ units of conductance, while in the symmetry-broken configuration (C), a single valley is filled with holes resulting in a total conductance of *G*_0_. (**D**) HF energies of the symmetric configuration (blue) and symmetry-broken configuration (orange) as a function of the total number of holes for the lowest subband (top) and second subband (bottom). (**E**) Transconductance ${dG/dV}_{SG}$ as a function of the upper split-gate voltage *V*_*SG*_ and carrier density in the leads as tuned by the back-gate voltage. The lower split gate and contact gates are kept constant at 4 and −7 V, respectively. (**F**) Conductance traces taken along the dashed lines in (E). (**G**) The ratio between the total width *w*_*T*_ and the width of the SB spin-polarized plateau *w*_1_ plotted as a function of carrier density. The black dashed line represents the relationship between the interaction strength *J* and the carrier density *n*, as expected from our model.

We control the width of the 1D channel electrostatically by applying 4 V to the lower split gate and by tuning the voltage applied to the upper split gate (*V*_*SG*_), resulting in the data presented in Fig. 2A at *B*_⊥_ = 0 T. The four-terminal conductance *G* is quantized, generating well-defined plateaus at multiples of 2*G*_0_ where *G*_0_ = *e*^2^/*h* is the conductance quantum, *e* is the electron charge, and *h* is the Planck’s constant. The double degeneracy of the conductance channels stems from the spin-valley–locked bands at *K* and *K*′. The observed conductance quantization is expected and well understood as 1D ballistic transmission subbands. We note that in previous reports, 1D transport in monolayer and few-layer TMDs had plateau-like features separated by conductance values of *G*_0_ (*19*–*23*), a behavior that was not fully understood. In addition to the expected 2*G*_0_> degeneracy, we observe additional features at *G*_0_ and 3*G*_0_ which are the focus of this paper (Fig. 3A).

Applying *B*_⊥_ between −500 and 500 mT, corresponding to the purple and gold traces respectively in Fig. 2A, the plateaus located at integer multiples of 2*G*_0_ occurring at *B*_⊥_ = 0 T evolve into plateaus with integer values of *G*_0_, indicating that the twofold degeneracy of the spin-valley–locked bands has been lifted. Figure 2B displays the conductance as a function of *B*_⊥_ and *V*_*SG*_ where regions of constant conductance are outlined by white dashed lines, demonstrating the robustness of the 1D subbands. To precisely chart the subband edges, Fig. 2C presents the transconductance of the 1D channel by taking the numerical derivative $dG/dV_{SG}$. Dark regions indicate constant conductance, and the bright lines represent the transition between two plateaus. A clear subband splitting is observed at magnetic fields as low as ≈250 mT. We also note that the additional *G*_0_ and 3*G*_0_ plateaus observed at *B*_⊥_ = 0 T do not split with *B*_⊥_, hinting toward the existence of a spin-valley–polarized state at zero magnetic field.

To understand our observations, we develop an anisotropic massive Dirac Fermion model (see the Supplementary Materials) described by the Hamiltonian

$${\hat{H}}_{\text{MDF}}=\frac{\Delta }{2}\sigma_{z}+\alpha V\left(y\right)I_{2}+\frac{\lambda }{2}\left(I_{2}-\sigma_{z}\right)+\hbar v_{F}\left(\tau k_{x}\sigma_{x}+\frac{v_{⊥}}{v_{F}}k_{y}\sigma_{y}\right)$$ (1)

where τ = ±1 for the *K* (*K*′) valley, Δ = 1.6 eV is the main bandgap, 2λ = 0.46 eV is the spin-orbit constant, ℏ*v*_F_ = 0.3927 eV⋅nm is the Fermi velocity, *I*_2_ is the two-by-two identity matrix, and σ_*i*_ (*i* = *x*, *y*, *z*) are the Pauli matrices. The above effective 2D Hamiltonian is valid for both low-energy conduction and valence band states near the band edges at the *K* and *K*′ valley and correctly accounts for the valley topology while including the bandgap and the spin-orbit interactions (*10*, *12*, *30*–*32*). The parameter *v*_⊥_ denotes the effective Fermi velocity in the direction perpendicular to the channel. Its value is a fitting parameter, and its departure from the value *v*_F_ is due to Coulomb interactions. The channel potential *V*(*y*) is multiplied by the lever arm parameter α = 1.2 meV/V (see the Supplementary Materials). We use this model to calculate the valence subband dispersions with *V*(*y*) obtained by solving the Laplace equation numerically for all experimental values of *V*_*SG*_ (see the Supplementary Materials).

Figure 2D shows the evolution of the first few subband edge minima as a function of *V*_*SG*_ at *B*_⊥_ = 0 T. Two examples of the potential landscape, when *V*_*SG*_ = 12 V and *V*_*SG*_ = 6.5 V, are plotted in the top of Fig. 2E with their respectively calculated subband dispersions in the bottom. With the assumption that the Fermi energy in the leads does not depend on the gate potentials, we pin the Fermi energy at the onset of the first subband at *V*_*SG*_ = 12 V [red dot in Fig. 2 (C to E)]. By using the position of the intersection of the second subband and the Fermi energy as a fitting target for the effective Fermi velocity *v*_⊥_, we find that the remaining calculated intersections agree well with the experiment [horizontal dashed lines in Fig. 2 (C and D)].

### Valley-polarized transport at zero magnetic field

This effective model captures well the conductance edges of doubly degenerate subbands, but it does not capture the features at *G*_0_ and 3*G*_0_ at *B*_⊥_ = 0 T, highlighted by red arrows in Fig. 3A. To do so, we include hole-hole interactions to the model using the Hartree-Fock (HF) approximation for two different scenarios of band filling as a function of the number of holes present in the transport channel (see the Supplementary Materials).

Our theoretical approach involves resolving the competition of single-particle and exchange energies close to the van Hove singularity characteristic for the density of states at the edge of 1D subbands, which is smeared out as a result of the finite length of our channel. This approach was previously discussed in the context of the 0.7 anomaly, a partial spin polarization effect reported in semiconductors such as gallium arsenide, and whose origin is still under debate (*33*–*40*). First, we consider the lowest subband from each valley. The subbands may be populated with holes in a valley-symmetric configuration, schematically represented in Fig. 3B, where each subband is filled with the same number of holes (*N*/2) and each contributes *G*_0_ units of conductance. Alternatively, in the symmetry-broken configuration, only one subband originating from one valley is filled with *N* holes leading to a total conductance of *G*_0_ (Fig. 3C). The corresponding energies $E_{HF}^{S}$ (HF energy of the symmetric configuration) and $E_{HF}^{SB}$ (HF energy of the symmetry-broken configuration) are calculated as

$$E_{HF}^{S}\left(N\right)=2T\left(N/2\right)+U\left(N\right)-2J\left(N/2\right)$$ (2)

$$E_{HF}^{SB}\left(N\right)=T\left(N\right)+U\left(N\right)-J\left(N\right)$$ (3)

where *T* is the total single-particle kinetic energy, *U* is the total direct interaction energy, and *J* the total exchange interaction energy. We note that the direct term *U*(*N*) is equal for both scenarios since it does not depend on the wave numbers of the subband states, only on their subband indices. Therefore the difference between these two total energies depends on the balance between the kinetic energy and the exchange energy. We find that these two energies depend differently on the total number of holes *N*. The total kinetic energy increases approximately quadratically with *N*, since the dispersion relation is approximately parabolic, whereas the total exchange energy increases quasi-linearly with *N* due to the short-range nature of the exchange. As a result, we expect strong exchange interaction effects at small *N*, while at larger *N*, the difference between the energies $E_{HF}^{S}\left(N\right)$ and $E_{HF}^{SB}\left(N\right)$ will be mostly due to the difference in the total kinetic energy. The top of Fig. 3D shows the HF energy of the symmetric configuration (blue line) and the broken-symmetry configuration (orange line) ignoring the direct term. We find that for the first ≈100 holes admitted into the system, the broken-symmetry configuration is lower in energy than the symmetric one. Thus, we find a spontaneous symmetry breaking brought about by the strong exchange interactions. However, as the total number of holes becomes larger than 100, a symmetry-broken configuration to symmetric configuration (SB-S) transition occurs, and the symmetric configuration becomes the ground state. Consequently, only one conduction channel is available for the first 100 holes, and the second conduction channel opens when the transition occurs, in qualitative agreement with experimental data.

A similar calculation can be performed for the second subband where the bottom of Fig. 3D shows the relevant HF energies for the symmetric (blue line) and symmetry broken configuration (orange line). As for the first subband, the SB configuration is the ground state at a low subband population and transitions to the S configuration as more holes are introduced. This transition occurs at a lower number of holes in the subband stemming from the generally weaker exchange interactions in the second subband and appears in experiment as a shoulder-like feature at 3*G*_0_. Furthermore, because of the even weaker exchange interactions at higher subbands, no symmetry-broken configuration exists, in agreement with experimental results.

The effect of interactions can be tuned by changing the hole density *n* of the WSe_2_ with the back-gate. In Fig. 3E, we plot the transconductance ($dG/dV_{SG}$) as a function of *n* and *V*_*SG*_ and observe that the widths of the conductance plateaus change with *n*. Focusing on the first two plateaus at *G*_0_ and 2*G*_0_, Fig. 3F shows traces taken at *n* = −1.21 × 10^12^ cm^−2^ and at *n* = −1.52 × 10^12^ cm^−2^ corresponding to the purple and green dashed lines in Fig. 3E, respectively, demonstrating the dependence of the width on *n*. We label the width of the SB spin-polarized plateau as *w*_1_ and the total width corresponding to the sum of the SB spin-polarized plateau and the S spin-unpolarized as *w*_*T*_. With *w*_*T*_ corresponding to the intravalley energy difference between the edge of the first and second subbands and *w*_1_ corresponding to the intervalley subbband energy difference, the degree of valley-spin polarization in the system can be expressed as the ratio *w*_*T*_/*w*_1_. To illustrate the tunability of this degree of polarization, the ratio *w*_*T*_/*w*_1_ as a function of *n* is plotted in Fig. 3G, where we confirm a similar trend to the one between *J* and *n* (see the Supplementary Materials); as the carrier density increases, the ratio between *w*_*T*_ and *w*_1_ also increases. This trend is a clear signature of the competition of single-particle and exchange energy scales. At sufficiently large densities, the former begins to dominate over the latter, resulting in the restoration of the Kramers symmetry and disappearance of the plateau with unit conductance. The overall trend, increasing *w*_*T*_/*w*_1_ with respect to increasing carrier density, demonstrates that the valley-spin polarization and hole-hole interactions are tunable in our device by controlling the back-gate voltage.

### Interaction-driven reconstruction and subband freezing at high magnetic field

**Fig. 4.**
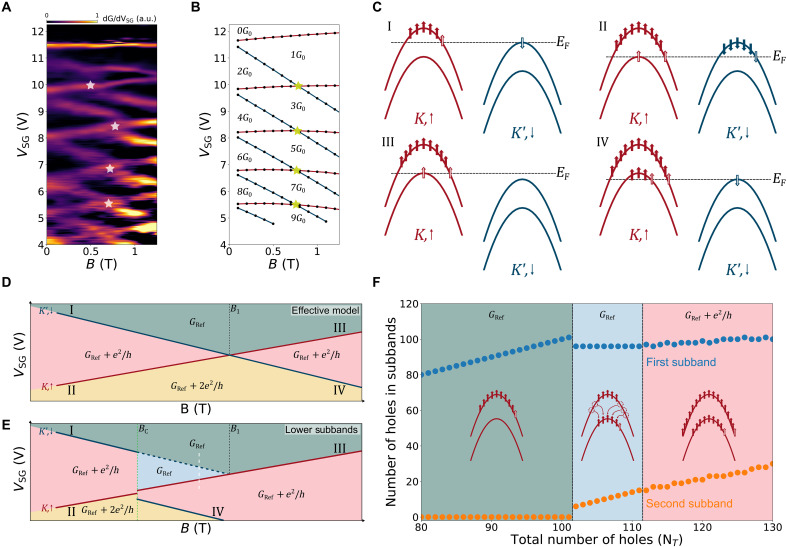
Interaction-driven reconstruction at high magnetic field. (**A**) 1D channel transconductance as a function of gate voltage and magnetic field. (**B**) Positions of the addition steps as a function of the split-gate voltage and the magnetic field calculated using the anisotropic massive Dirac Fermion mode and using $g_{e,Z}=-2$ and $g_{h,Z}=2.6$. White and gold stars in (A) and (B) indicate where subband crossings occur. (**C**) Depictions of the possible single-particle subband configurations near a subband crossing. Filled arrows denote the holes already residing in the channel, and empty arrows denote the next holes being added to the system. (**D** and **E**) Schematic of the conductance as a function of *B*_⊥_ and *V*_*SG*_ in the single particle picture (D) and when considering hole-hole interactions (E). The transition lines labeled by I to IV correspond to the configurations depicted in (C). The colored areas are labeled by their respective conductance values. (**F**) Occupation of the first two subbands at *K* as a function of the total number of holes in the system obtained from HF calculations. This calculation is performed along the white dashed line in (E).

The application of a magnetic field beyond 0.5 T gives additional insights on the role of hole-hole interactions and how they depend on the subband population, as indicated in Fig. 4A. White stars in Fig. 4A indicate intervalley subband crossings where the spin-valley Zeeman splitting is equal to the intravalley subband spacing. We observe intersections with minute kinks for the third and higher crossings; however, clear discontinuities are evident for the first two (*41*–*43*). To understand these observations, we include a magnetic field in the anisotropic massive Dirac Fermion model by adding to the Hamiltonian (i) a Zeeman term $\frac{1}{2}g_{e\left(h\right),Z}\mu_{B}B$ where $g_{e,Z}=2$ is the Landé factor of a free electron and μ_B_ is the Bohr magneton and (ii) diamagnetic effects by replacing the momentum $\vec{k}$ with the generalized momentum ${\vec{p}}_{i}=\vec{k}+e\vec{A}$ where *e* is the electron charge and $\vec{A}$ is the vector potential. The anisotropic hole Landé factor $g_{h,Z}$ is a fitting parameter. Choosing $g_{h,Z}=+2.6$ yields good agreement between the theory and the experiment as indicated in Fig. 4B. Using this value of $g_{h,Z}$, we can fit the subband splitting in Fig. 2C (or Fig. 4A) and extract the hole valley g-factor $g_{h,v}$ for the first four subbands from $\Delta E=\left(g_{h,v}+g_{h,Z}\right)\mu_{B}\Delta B$. We find that $g_{h,v}^{1}=13\pm 3$, $g_{h,v}^{2}=21\pm 5$, $g_{h,v}^{3}=40\pm 10$, and $g_{h,v}^{4}=40\pm 10$. This suggests that as the 1D confinement and the interactions become stronger, the valley g-factor is reduced, distinct from 2D transport in monolayer TMDs (*24*, *44*). The reason behind this discrepancy is the 1D confinement potential. Hole-hole interactions enhance $g_{h,v}$ because of exchange, while confinement weakens $g_{h,v}$ because it governs the energy quantization. In other words, the stronger the confinement is, the stronger the magnetic field is needed to form cyclotron orbits, leading to a smaller $g_{h,v}$. The extracted valley g-factors are a result of this competition.

We can qualitatively understand the nature of the crossings by using the HF approximation to calculate (i) the total energies of the various subband filling configurations as a function of the magnetic field and (ii) the number of holes in each subband as a function of the total number of holes for a fixed magnetic field. Figure 4C shows the four different configurations relevant in the system, while their corresponding transition lines in the *B*_⊥_-*V*_*SG*_ plane are schematically represented in Fig. 4D. This representation corresponds to the single particle picture and does not include hole-hole interactions which is similar to the case of the third and higher subbands where interactions are less important. In the green region, a certain number of subbands are already populated and contribute to the conduction, which we label as *G*_*Ref*_. We see that as *V*_*SG*_ is decreased at low magnetic field, the subband filling follows configuration I and then configuration II, first filling the K′,↓ band with a step of *G*_0_ and then the second K,↑ band with another *G*_0_ conductance step. Above the crossover point *B*_1_, the spin-valley Zeeman energy makes it energetically favorable to first fill the K,↑ band and then K′,↓ band. In experiment, for the lower subbands where interactions are stronger, we detect a discontinuity in the crossover region at a critical magnetic field labeled *B*_c_. This effect is schematically shown in Fig. 4E. Following the white dashed line in this case, the system follows the subband filling sequence presented in Fig. 4F, where we calculate the energy of the first two subbands at *K* as a function of the number of holes in the system. The first transition, between the green and blue regions, corresponds to a sudden redistribution of holes within the subbands in which competition between kinetic and interaction energies transfers ≈5 holes from the first to the second subband. This transition is not observed in our experiment as it only corresponds to a redistribution of holes and not to any additional conducting subbands; therefore, there is no change in conductance. Within the blue region, the first subband population is frozen, and further holes only fill the second subband. Transitioning to the pink region, it is energetically favorable to fill both bands leading to a *G*_0_ increase in conductance. This sudden reallocation (transition from green to blue region) is due to the fact that the exchange energy is extremely local in *k*-space leading to a redistribution of the holes toward the vicinity of the subband minima, although the promotion of the last added holes to the second subband costs kinetic energy. Furthermore, adding another hole to an already substantially filled lowest subband is not favorable energetically, since the new hole will be far (in *k*-space) from the band minimum, while adding it to the slightly filled second subband brings it much closer to other holes. Thus, only one conduction channel exists, involving the second subband only. As that subband is filled with subsequent holes, they are added further away from the second subband minimum, and the advantage of the exchange energy becomes less pronounced. At this point, the holes are added to either subband in sequence, creating two conduction channels, which shows as a jump in conductance. This interaction-driven subband filling sequence leads to the particular discontinuities measured in Fig. 4A for the lower subbands in which interactions are stronger.

### Configuration-interaction picture

**Fig. 5.**
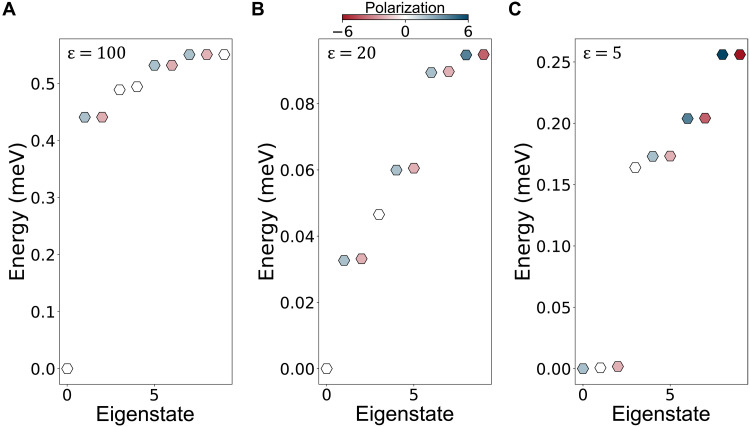
Configuration-interaction picture. Energies of six interacting holes at *B*_⊥_ = 0 T as a function of the lowest energy configurations. The color scale represents the spin-valley polarization of the configuration. The energies of the various configurations are calculated for different dielectric constants where ε = 100 in (**A**), ε = 20 in (**B**), and ε = 5 in (**C**).

We have demonstrated that a complete understanding of hole-hole interactions is necessary to explain all experimental features of the system. In systems of interacting fermions, we typically deal with three aspects of interactions: direct, exchange, and correlations. In our theoretical model, we used the HF approach, which accounts for the first two aspects but underestimates the third. As we have demonstrated, the exchange interactions stabilize the spin-valley–polarized phases. However, the inclusion of correlations lowers the energy of low-spin phases (*45*). Thus, we need to show that the spin-valley–polarized, symmetry-broken phases remain ground states of our system even if the correlations are accounted for. We do this using the configuration interaction approach, which treats all three aspects of interactions on equal footing. However, its computational complexity makes it impossible to apply it to a system with the dimensions of this experiment; therefore, we consider a 1D channel with a width of 60 nm and a length of 150 nm. In Fig. 5, we show the results of calculations of energies of six interacting holes at zero magnetic field. We find that the nature of the ground state of six holes strongly depends on the strength of Coulomb interactions, tuned by the choice of the dielectric constant ε. For a weakly interacting system (ε = 100; Fig. 5A), the ground state is the fully symmetric singlet configuration, with three holes in each valley. This state is separated from the excited states by a gap corresponding to the intersubband spacing. With interactions of moderate strength (ε = 20; Fig. 5B), the partially polarized states (color symbols) are separated from the unpolarized symmetric ground state by a much smaller gap. Last, with strong interactions (ε = 5, close to experimental conditions; Fig. 5C), the partially polarized, symmetry broken states and the symmetric singlet state are at similar energies, and a partially polarized state appears to take over as the global ground state of the system. We stress that in this strongly confined system, the kinetic energy quantization is much stronger than in the channel probed experimentally, making it more difficult for the partially polarized states to stabilize. Moreover, in this calculation, the direct, exchange, and correlations are screened by the same dielectric constant ε, while our experimental results demonstrate that the degree of screening depends on the aspect of interactions, resulting in the exchange being stronger than direct (see the Supplementary Materials). Thus, our symmetry broken, polarized ground state is found to be robust against correlations, although the domains of its stability in parameter space may be smaller than our HF approach predicts.

## DISCUSSION

We have elucidated the role that strong exchange interactions and many body correlation effects play in quantum transport in TMDs and specifically how they affect the spin and valley degrees of freedom. The high quality of our device allows for ballistic transport in a monolayer WSe_2_ 1D channel, resulting in several conductance quantization steps. We demonstrate that the degeneracy of each transport subband in this material is neither fourfold (containing both spin and valley degrees of freedom) nor single (as previously reported) but twofold due to spin-valley locking. By tuning the carrier density, we control the interaction strength and can establish a fully spin-valley–polarized transport channel at zero magnetic field with *G*_0_ conductance. The origin of this spontaneous spin-valley polarization is a competition between kinetic and exchange energies leading to a breaking of the valley symmetry in the system. Using the HF approximation to include interactions, we demonstrate this symmetry breaking and furthermore predict discontinuities in the 1D magneto-transport spectrum which are corroborated by experiment. These findings demonstrate marked differences between TMDs and other quantum materials and can have deep consequences in the emerging field of valleytronics, where it is essential to understand the role of interactions between particles in different valleys to control the valley degree of freedom.

## MATERIALS AND METHODS

### Device fabrication

Bulk crystals were exfoliated on a silicon substrate with 285 nm of thermal oxide to obtain the monolayer WSe_2_, hBN, and few-layer graphite flakes used to fabricate the heterostructure illustrated in Fig. 1. Using an optical microscope, flakes with suitable thicknesses, geometry, and uniformity were identified. Their topographic properties were then confirmed using an atomic force microscopy (Bruker Dimension Icon) in tapping mode (SCANASYST-AIR). The heterostructure was assembled using a dry transfer technique aided by a polypropylene carbonate transfer polymer and operating with temperatures ranging from 40° to 90°C. First, a bottom hBN flake measuring 24 nm in thickness was picked up and then used to pick up a 4-nm-thick flake of graphite. This stack of flakes was deposited on an intrinsic silicon substrate covered by 285 nm of thermally grown oxide. An electrical contact was made to the graphite flake by first using a selective reactive ion etch using CF_4_ and O_2_ plasmas to etch a window in the hBN flake followed by metal deposition. Electron beam lithography and electron beam metal evaporation were used to pattern metallic contacts (2 nm of chromium + 8 nm of platinum) on top of the hBN. To remove any residue that may have accumulated on the surface of the contacts and the hBN during the fabrication process, the sample was annealed in a vacuum chamber (10^–7^ torr) at 300°C for 30 min. In addition, an atomic force microscope (AFM) operating in contact mode was used to displace any remaining residue residing on the contacts and hBN. A top hBN flake measuring 25 nm in thickness and monolayer WSe_2_ were sequentially picked up, AFM cleaned, and dropped off onto the electrical contacts. After encapsulation, the heterostructure was once again annealed in a vacuum furnace following the same recipe as described earlier. The contact top gates (*V*_*CG*_) and split gates (*V*_*SG*_) were patterned using electron beam lithography and metallized using electron beam deposition. Additional optical micrographs of the various device fabrication steps are provided in the Supplementary Materials.

### Experimental setup

Transport measurements were performed in a Bluefors LD250 dry dilution refrigerator, equipped with an 8 T magnet, where measurements were conducted at the base temperature of 10 mK, unless otherwise noted. Standard four terminal dc conductance measurements were performed using a Keysight source measuring unit SMU2901a and an Agilent digital multimeter with a constant bias of 250 μV applied between the source and the drain contacts. All gates (split gates, contact gates, and back-gate) were independently tuned using individual Keysight source measuring units (SMU2901a/SMU2902b). See the Supplementary Materials for the exact measurement configuration and individual contact characterization.

## Supplementary Materials

### This PDF file includes:

Supplementary Text Figs. S1 to S8 Ref
