# Supplementary material for: Valley-spin polarization at zero magnetic field induced by strong hole-hole interactions in monolayer WSe2
Source: Sci Adv. 2025 May 7;11(19):eadu4696. doi: 10.1126/sciadv.adu4696 (PMC12057658; doi:10.1126/sciadv.adu4696)
Supplement: Supplementary file 1 — Supplementary Text Figs. S1 to S8 Ref [file sciadv.adu4696_sm.pdf]

Science Advances
AAAS

Supplementary Materials for

Valley-spin polarization at zero magnetic field induced by strong hole-hole interactions in monolayer WSe_2

Justin Boddison-Chouinard *et al.*

Corresponding author: Adina Luican-Mayer, luican-mayer@uottawa.ca;
Louis Gaudreau, louis.gaudreau@nrc-cnrc.gc.ca

Sci. Adv. **11**, eadu4696 (2025)
DOI: 10.1126/sciadv.adu4696

This PDF file includes:

Supplementary Text
Figs. S1 to S8
References

I. DEVICE FABRICATION AND CONTACT CHARACTERIZATION

The contact resistance of individual contacts as a function of the contact gate voltage at 10 mK is obtained following the circuit diagram in Fig. S2a. The voltage probes of a 4-point measurement setup are placed on the contact of interest and an adjacent contact. Current is forced to flow from the contact of interest to a third contact with all other contacts floating. The measured resistance is therefore a sum of 1) the contact resistance of the contact connected to the voltage probe and the current source, and 2) a small contribution from the channel, therefore this technique slightly overestimates the contact resistance. The contact resistance is measured as a function of the contact gate directly above it, while the other contact gate is kept constant at -7 V to ensure all contacts in the circuit are activated. The back-gate is fixed at -3 V to activate the channel and reduce the contribution originating from channel resistance. The split-gates are both set to -0.5 V to avoid the formation of a 1D channel. Using this technique, the following contact resistances are extracted (Fig. S2 c-g): $R_{LC1} = 711\ \Omega$ and $R_{LC2} = 982\ \Omega$ at V_{LCG} = -8 V, and $R_{RC1} = 5.31$ kΩ, $R_{RC2} = 4.55$ kΩ, and $R_{RC3} = 2.01$ kΩ at V_{RCG} = -7 V. For the 4-point measurements presented in the main manuscript, LC1 was connected to the current source, RC2 was connected to ground, and LC2 and RC3 were connected to the voltage meter. To further demonstrate the quality of the electrical contacts, Fig. S2h shows a linear relationship between the measured current and applied bias voltage between contacts LC1 and RC2 with a total resistance of 5.9 kΩ indicating that ohmic contacts are achieved.

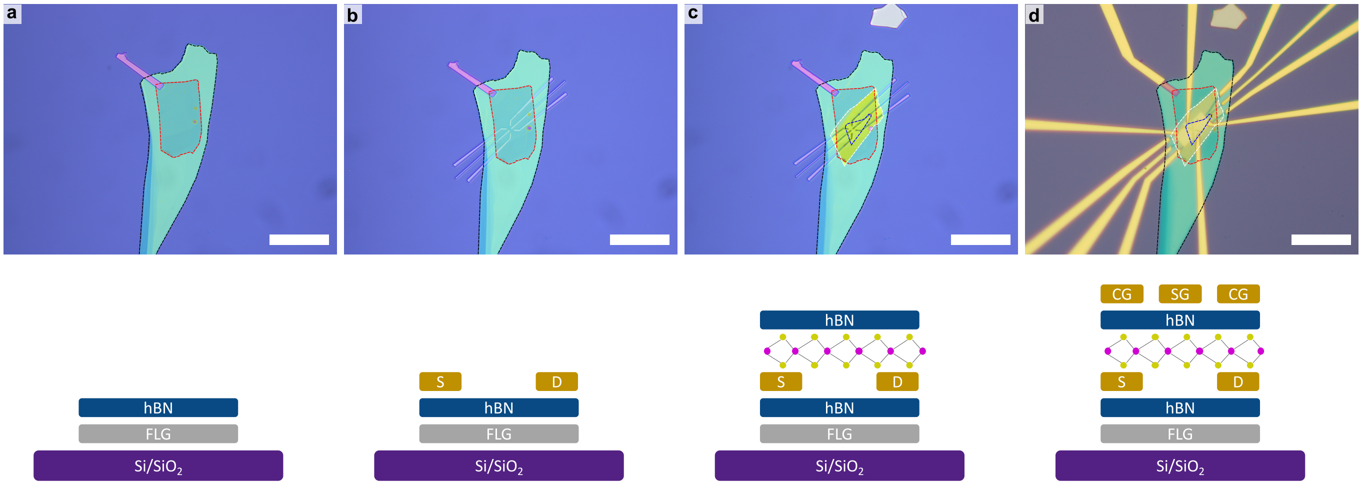

FIG. S1. **Device fabrication.** **(a-d)** Optical micrographs (top) and cartoon schematics (bottom) showing the assembly process of the heterostructure device discussed in the main manuscript. In the optical micrographs, the individual flakes are outlined by the following colors: few-layer graphite = red, bottom hBN = black, monolayer WSe_2 = blue, and top hBN = red.

II. THEORETICAL MODEL

A. Massive Dirac Fermion Model at Zero Magnetic Field

1. *Hamiltonian and parametrization*

To describe our system and understand our observations, we begin with the massive Dirac Fermion model, in which the Hamiltonian is written in the two-band basis as

$$\hat{H}_\tau = \begin{bmatrix} \frac{\Delta}{2} + \alpha V(y) & \hbar v_F(\tau\hat{k}_x - i\hat{k}_y) \\ \hbar v_F(\tau\hat{k}_x + i\hat{k}_y) & -\frac{\Delta}{2} + \lambda + \alpha V(y) \end{bmatrix}. \quad \text{(S1)}$$

Here, τ is the valley index, so that $\tau = 1$ corresponds to the valley K, and $\tau = -1$ denotes the valley K'. The parameter Δ defines the bandgap, while λ corrects the top of the valence band due to the spin-orbit interaction. The parameter v_F is the Fermi velocity and $\hbar$ is the Dirac's constant. Further, $V(y)$ is the channel potential (discussed

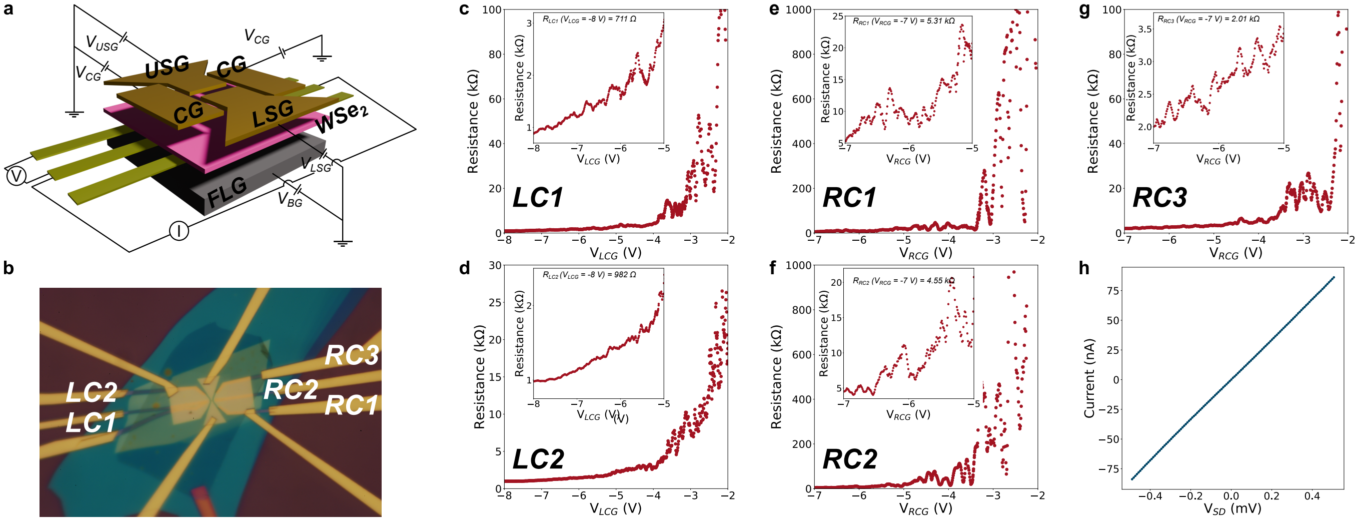

FIG. S2. **Contact resistance and quality.** **(a)** Schematic illustration of a 3-terminal measurement used to measure the contact resistance of individual contacts. Contact LC1 can be measured using the wiring example provided in this schematic. We note that for the 4-terminal measurement used throughout the article, LC2 was connected to the source, RC3 was the drain, and LC1 and RC2 were used as voltage probes. **(b)** Optical micrograph of the device where individual contacts are labelled. **(c-g)** Contact resistances of each contact as a function of the contact gates measured at 10 mK. When measuring the contact resistance of a contact located on the left (right) side, the right (left) contact gate is fixed at -7 V. V_{BG} = -3 V and V_{LSG} = V_{USG} = -0.5 V. The inset focus on the low resistance range. **(h)** 2-point IV measurement using LC2 as the source and RC2 as the drain.

in SI III), and α is the lever arm translating the voltages into the energy scale (discussed in SI IV). Lastly, $\hat{k}_x$ and $\hat{k}_y$ are the components of the gradient operator, $\hat{\vec{k}} = -i\vec{\nabla}$.

The above Hamiltonian is formulated in the electron edge, i.e., it accounts for the coupling of the electronic states in the conduction band and the electronic states in the valence band. It is consistent with that given in Ref. (12). Here we account for the spin-valley locking phenomenon and consider only two bands per valley, corresponding to the spin subspace forming the valence band edge. Specifically, we take only the spin-up subspace in the valley K, and the spin-down subspace in the valley K'. This is why in our treatment the spin-orbit parameter λ modifies the top of the valence band identically in each valley. The parameters for WSe_2 are also taken from Ref. (12) and are $\Delta = 1.6$ eV, $\lambda = 0.23$ eV, and $\hbar v_F = 0.3927$ eV·nm.

2. *Computational procedure*

We assume that the one-dimensional channel is sufficiently long for us to treat it as translationally invariant along the x axis, while the channel confinement $V(y)$ generated by the gates has to be taken into account explicitely along the y axis. Since $V(y)$ is in a numerical form, we look for the eigenenergies and eigenstates of the Hamiltonian (S1) in a numerical computational procedure. To this end, we choose the following basis set:

$$\langle \vec{r}|n,k\rangle = \psi_{n,k}(x,y) = \frac{1}{\sqrt{L}} \exp\left(ikx\right) \sqrt{\frac{2}{W}} \sin\left[\frac{n\pi}{W}\left(y + \frac{W}{2}\right)\right]. \tag{S2}$$

These basis functions are products of the function in the x direction (with translational symmetry) and the y direction (the confinement direction).

In the x direction we choose a plane wave function reflecting the translational invariance. Further, we assume periodic boundary conditions on our one-dimensional channel, which require

$$\exp(ik0) = \exp(ikL), \tag{S3}$$

and thus discretize the wave vector $k = 2\pi m/L$, with the integer $m = 0, \pm1, \pm2, \ldots$. We note that the Hamiltonian (S1) conserves the wave vector k.

In the y direction we choose to enclose our potential $V(y)$ in an infinite quantum well of width W. Our basis of sines is simply the basis of eigenstates of that quantum well, with the origin chosen in the middle of the channel. The basis functions are enumerated by the integer $n = 1, 2, \ldots$. We take the width $W = 563.2$ nm, slightly narrower than the spatial extent of the calculated potential profiles (Sup. III). This choice of W is made by finding the minimal region encompassing the spatial variation of the potential, i.e, beyond which the potential becomes flat (coordinate-independent).

As the MDF model is a two-band approach, we seek the single-particle eigenstates in the dimer form

$$|k, S\rangle = \begin{bmatrix} \sum_{n=1}^{N_B} A_n^{k,S} |n, k\rangle \\ \sum_{n=1}^{N_B} B_n^{k,S} |n, k\rangle \end{bmatrix}, \tag{S4}$$

where S is the subband index, the coefficients $A_n^{k,S}$, $B_n^{k,S}$ form the eigenvectors of the Hamiltonian (S1), and N_B is the basis size (we take $N_B = 150$). The above eigenstates, as well as the corresponding single-particle energies $E_{S,k}$ are obtained by formulating the Hamiltonian (S1) in a matrix form in our basis and diagonalizing it numerically.

3. *Single-hole states in the channel*

Figure S3a shows the dispersion calculated for the gate potential generated with the gate voltage $V_{USG} = 12$ V. We focus on the valence band states only. The single-particle energies are plotted as a function of the wave number k, i.e., the index of the plane wave along the channel. In the hole language the energy increases towards the bottom of the graph, and therefore the topmost trace corresponds to the lowest hole subband confined in the channel, $S = 1$. As we go downwards in energy, we encounter the subsequent subbands, $S = 2, 3, \ldots$. We find that the dispersions for the valleys K and K' are exactly degenerate.

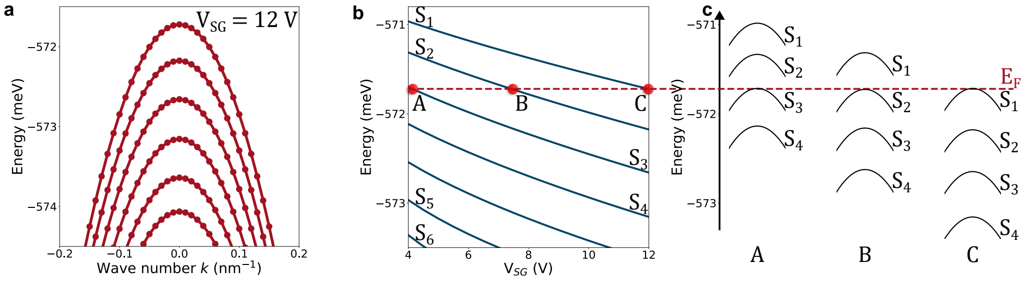

FIG. S3. **Single-particle Dispersion.** **(a)** Dispersion for the one-dimensional channel with the confinement potential calculated for $V_{USG} = 12$ V. **(b)** Evolution of the edges of several lowest hole subbands as a function of the gate voltage V_{USG}. **c** Sketch of the band dispersion alignment at points A,B, and C. The red dashed line marks the Fermi energy.

Assuming that the Fermi energy in the leads do not depend on the gate potentials, as the potential V_{USG} is made less positive, the hole subbands shift and cross the fixed Fermi level one by one. From Fig. S3a it is evident that this crossing involves the bottom of the subband, i.e., the state at $k = 0$, because this is the lowest-energy hole state within each subband. In the choice of energy axis consistent with Fig. S3a, gradual decrease of the voltage V_{USG} will result in a shift of all the subbands upwards in energy. In that evolution, whenever the Fermi energy aligns with the bottom of any new subband, a new conduction channel opens, resulting in a step in conductance.

Figure S3b shows the evolution of several of the lowest channel subband edges (i.e., subband energies at $k = 0$) as a function of the voltage V_{USG}. The red dashed line shows the Fermi energy, which we have chosen to reproduce the first conductance step occurring at $V_{USG} = 12$ V. This is consistent with the experimental data shown for $B = 0$. We note that these subband edges are doubly degenerate owing to the valley degeneracy. Therefore, at $V_{USG} = 12$ V (diagram C in Fig. S3c) we would expect an onset of conductance of *two* units, i.e., by $2e^2/h$, because two conduction channels become simultaneously available for the holes. The next onset, by another *two* units, would occur at the voltage $V_{USG} \approx 7.5$ V (diagram B in Fig. S3c). The third step, again by *two* units, is expected at $V_{USG} \approx 4$ V (diagram A in Fig. S3c). This is, however, not what we see experimentally. It is evident from experiment that the first conductance step, occurring at $V_{USG} = 12$ V, is only by *one* unit of e^2/h. The second step follows at $V_{USG} \approx$ 11.2 V and is also by one unit of conductance. The third and fourth steps, one unit each, occur at $V_{USG} \approx 10.3$ V and 9.8 V, respectively. The fifth and sixth signature occurs at $V_{USG} \approx 8.3$ V and 6.9 V, respectively, and at $B = 0$ each of these steps is by $2e^2/h$.

We therefore draw two conclusions: first, a mechanism exists for removing the degeneracy of the two lowest pairs of subbands, which has been explained at length in the main manuscript, and second, our single-particle model appears to overestimate the gaps between subsequent subbands, causing them to cross the Fermi energy for voltages V_{USG} much less positive than those recorded in experiment. Therefore, we now turn to including the interactions in our model approach.

B. Hartree-Fock Model of Interacting Holes in the One-Dimensional Channel

1. *The Hamiltonian*

We now account for the Coulomb interactions among the holes populating the single-particle levels. Let us denote the creation (annihilation) operator of a hole on the level $\gamma = \{S, k, \sigma\}$ by c^+_γ (c_γ). In our composite orbital index γ we have added the spin-valley index σ to the quantum numbers S, k describing the single-particle orbital. We note that we only need one spin-valley index owing to the spin-valley locking, as explained in the discussion of the Hamiltonian (S1). In consequence, $\sigma =\uparrow$ will denote a spin-up hole in the valley K, and $\sigma =\downarrow$ will denote a spin-down hole in the valley K'.

In the language of the creation and annihilation operators, the Hamiltonian of interacting holes in our one-dimensional channel is written as

$$\hat{H}_I = \sum_\gamma E_\gamma c^+_\gamma c_\gamma + \frac{1}{2} \sum_{\gamma_1,\gamma_2,\gamma_3,\gamma_4} \langle \gamma_1, \gamma_2 | V | \gamma_3, \gamma_4 \rangle c^+_{\gamma_1} c^+_{\gamma_2} c_{\gamma_3} c_{\gamma_4}, \tag{S5}$$

where $\langle \gamma_1, \gamma_2 | V | \gamma_3, \gamma_4 \rangle$ denote the matrix elements of the Coulomb interaction calculated in the basis of single-particle orbitals.

2. *The Hartree-Fock approach*

To understand a system containing many holes, we employ a simplified Hartree-Fock (HF) model, where we calculate the total energies (expectation values of the Hamiltonian $\hat{H}_I$, or HF energies) of several candidate hole configurations. The ground state of the system of N holes is identified as the configuration with the lowest HF energy.

The HF treatment formally starts with writing down a candidate configuration in the form of a single Slater determinant. In the most general terms, we assume that we populate different subbands in both valleys with holes up to a certain state (indexed by momentum $k^{(S,\sigma)}_{MAX}$), which depends on the subband index and the valley. Our configurations therefore takes the following form

$$|\Psi\rangle = \prod_{S=1}^{S^\uparrow_{MAX}} \prod_{k=-k^{(S,\uparrow)}_{MAX}}^{k^{(S,\uparrow)}_{MAX}} c^+_{S,k,\uparrow} \prod_{S'=1}^{S^\downarrow_{MAX}} \prod_{k=-k^{(S,\downarrow)}_{MAX}}^{k^{(S,\downarrow)}_{MAX}} c^+_{S',k,\downarrow} |0\rangle, \tag{S6}$$

where $|0\rangle$ is the vacuum state. If we traverse the set of all *occupied* configurations S, k, σ by the index γ, the HF energy corresponding to the above configuration is

$$E_{HF}(\Psi) = \langle \Psi | \hat{H}_I | \Psi \rangle = \sum_\gamma E_\gamma + \frac{1}{2} \left[\sum_{\gamma,\gamma'} \langle \gamma, \gamma' | V | \gamma', \gamma \rangle - \langle \gamma, \gamma' | V | \gamma, \gamma' \rangle \right]. \tag{S7}$$

In this energy, the first term is the total single-particle energy of the holes. The second term accounts for direct and exchange interactions, represented respectively by the first and second Coulomb element within the sum.

The addition energy of the $(N+1)^{\mathrm{st}}$ hole to a system of N interacting holes, which occupy the states from the set $\{\gamma\}$, will depend on the index $\gamma_1 = \{S_1, k_1, \sigma_1\}$ of the single-particle orbital to which that hole is added. This addition energy is equivalent to the HF quasiparticle energy for the orbital γ_1 in the presence of the N holes and can be expressed as

$$E_{HF}(\gamma_1) = E_{\gamma_1} + \sum_{\gamma} \langle \gamma, \gamma_1 | V | \gamma_1, \gamma \rangle - \langle \gamma, \gamma_1 | V | \gamma, \gamma_1 \rangle. \tag{S8}$$

The addition energy of the hole added to the orbital γ_1 will consist of the single-particle energy of that orbital (the first term) and the selfenergy $\Sigma(\gamma_1)$, which accounts for all repulsive direct interaction elements with the N resident holes (the second term), and all attractive exchange interaction terms with these holes (the third term).

3. *Coulomb matrix elements*

To complete the HF model, we have to specify the Coulomb direct and exchange matrix elements. Since we have the single-particle orbitals in the form of Eq. (S4), we can obtain these matrix elements by direct integration of the Coulomb potential. However, the single-particle basis set $N_B = 150$ makes it prohibitively expensive to apply a full numerical calculation of a set of matrix elements required for tens of holes. This is the case even if we account for the fact that in Eq. (S4) most of the coefficients A are negligibly small, leaving us only with large amplitudes B for half of that state. Instead, we notice that the hole subbands have a nearly parabolic dispersion close to the band minima, as is evident from Fig. S3a, and the channel confinement potential (Sup. III) is also approximately parabolic near to its bottom. We will therefore approximate the hole wave functions by

$$\langle \vec{r} | k, S \rangle = \frac{1}{\sqrt{L}} \exp(ikx) f_S(y), \tag{S9}$$

where the subband functions f_S are taken in the form of the eigenstates of a one-dimensional harmonic oscillator. These functions are scaled by a distance parameter l, i.e., the oscillator length. This length is usually expressed by the particle effective mass and the frequency of the harmonic confinement. In our approach we will extract the values of l from the channel confinement profiles by noticing that at the distance l from the origin, the value of the harmonic potential energy is equal to the ground-state energy above the potential floor (it is a classical turning point). We note further that the wave number k in our single-particle functions is measured from the valley momentum, K and $-K$ for the valley K and K', respectively. This will be important in distinguishing between the intravalley and intervalley matrix elements.

The direct matrix element calculated with the $1/r$ Coulomb potential takes the form

$$\langle \gamma, \gamma_1 | V | \gamma_1, \gamma \rangle = \frac{e^2}{4\pi\epsilon_0\epsilon_r L^2} \int_0^L dx_1 \int_0^L dx_2 \int_{-\infty}^{\infty} dy_1 \int_{-\infty}^{\infty} dy_2 \frac{|f_S(y_1)|^2 |f_{S_1}(y_2)|^2}{\sqrt{(x_1-x_2)^2 + (y_1-y_2)^2}}, \tag{S10}$$

with e, ϵ_0, and ϵ_r being the electron charge, the vacuum electrical permittivity, and the dielectric constant of the material, respectively. The subband index S (S_1) originates from the compound index γ (γ_1). We find that the direct term depends exclusively on the subband indices. It does not depend on the wave numbers k, k_1 of the particles involved. Moreover, for the same subband indices, the values of the intervalley and intravalley elements are identical.

As for the intravalley exchange element, we have

$$\begin{aligned} \langle \gamma, \gamma_1 | V | \gamma, \gamma_1 \rangle = & \frac{e^2}{4\pi\epsilon_0\epsilon_r L^2} \int_0^L dx_1 \int_0^L dx_2 \\ & \times \int_{-\infty}^{\infty} dy_1 f_S(y_1) f_{S_1}(y_1) \int_{-\infty}^{\infty} dy_2 f_S(y_2) f_{S_1}(y_2) \frac{\exp\left[i(k-k_1)(x_1-x_2)\right]}{\sqrt{(x_1-x_2)^2 + (y_1-y_2)^2}}. \end{aligned} \tag{S11}$$

This element depends on both the subband indices and the wave numbers of the states involved. The intervalley exchange element is zero because of the spin-valley locking: holes in opposite valleys have opposite spins, which breaks the selection rules for the exchange interaction.

All matrix elements are scaled by the dielectric constant ε_r appropriate for our sample. Optical studies of multilayer WSe_2 samples reveal $\varepsilon_r \approx 6.24$ (Ref. 46), while density-functional calculations predict $\varepsilon_r \approx 7.2$ for a monolayer and $\varepsilon_r \approx 8.1$ for a bulk WSe_2 material (47). Furthermore, both experimental (48) and theoretical (47, 49) treatments show that ε_r depends not only on the number of monolayers, but also on the material encapsulating the sample (in our case, hBN). In Ref. (49) it is shown that for hBN-encapsulated monolayer systems, it is actually more correct to assume ε_r equal to that of the dielectric, in our case the value of 3.5. As is evident, it is not clear which value is the correct one, particularly in conditions where nearby gates and holes in the two-dimensional gas in the leads may screen some aspects of the Coulomb interactions. As a result, we will treat ε_r as a fitting parameter and discuss in detail the dependence of our results on its specific value.

C. Addition Energies of Holes in the Interacting System and the Anisotropic Massive Dirac Fermion Model

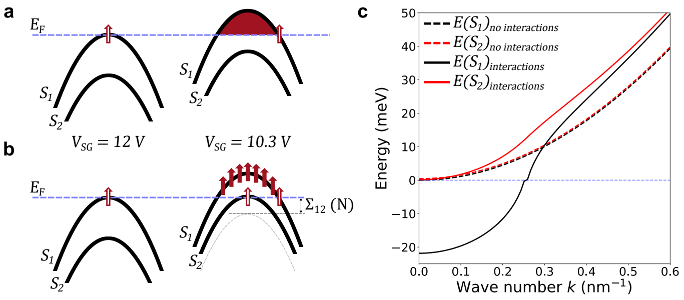

FIG. S4. **Effect of Coulomb interactions on the subband dispersion.** Alignment of the hole subbands in the K valley (K' valley behaves identically) at gate voltages $V_{USG} = 12$ V (left) and $V_{USG} = 10.3$ V (right) based on **(a)** the single-particle model without interactions and **(b)** model accounting for Coulomb interactions. **(c)** Energies of the two lowest subbands as a function of the wave number k. Dashed lines show the bands calculated with the single-particle Hamiltonian without interactions for the gate voltage $V_{USG} = 10.5$ V. Solid lines show the addition energies, i.e., the subband energies renormalized by direct and exchange interactions with $2N = 126$ resident holes, N holes per valley. Blue dashed lines show the Fermi energy.

1. *Schematic Picture*

In Fig. S4a we show schematically the alignment of hole subbands for the noninteracting model for the gate voltage $V_{USG} = 12$ V (left) and $V_{USG} = 10.3$ V (right). These subbands are plotted for the valley K, with their K' equivalents behaving identically. These voltages are chosen as they correspond to the onset of the lowest subband and the second subband, respectively (with the proper account for the valleys). In our noninteracting model, we have chosen the Fermi energy (represented by the blue line in Fig. S4a to coincide with the lowest hole subband at $V_{USG} = 12$ V. This accounts for the first conductance step, corresponding to the addition of the first hole, denoted schematically by the hollow red arrow, into the channel. However, when considering the Massive Dirac Fermion model, at the gate voltage $V_{USG} = 10.3$ V our second subband is not low enough in energy to coincide with the Fermi energy. The step heralding the opening of the second subband for conduction is expected only at $V_{USG} = 7.5$ V, as is evident from Fig. S3b.

Figure S4b shows the analogous subband alignment for the case accounting for interactions. The left panel is identical to the case in (a), because we are adding the first hole to the system (hollow red arrow), and there are no other holes to interact with. On the other hand, for the voltage $V_{USG} = 10.3$ V, we will now expect the conductance step, i.e., a hole being successfully added onto the second subband (hollow red arrow). Thus, the *quasiparticle* second subband (the solid line) will now be lowered in energy relative to the *noninteracting* second subband (dashed line) by the selfenergy $\Sigma_{12}(N)$, describing the interaction of the new hole with all N holes (red arrows) already present in the lowest subband. As is evident from our HF model, the holes being in the same valley and having the same spin will interact via exchange, which is attractive (lowers the quasiparticle energy). This mechanism allows us to find a quantitative correspondence of our simple model with the experimental data.

2. *Calculation of the selfenergy*

We assume that the lowest subband has been filled with $2N$ holes, N holes per valley, in the symmetric configuration. Indeed, in the experimental data we see that the onset of the second subband appears for voltages significantly lower than the phase transition from the polarized to the unpolarized lowest subband. As explained before, this transition occurs at $V_{USG} = 11.1$ V, i.e., the conduction step from e^2/h to $2e^2/h$. The selfenergy appearing in the addition energy of the hole to the second subband in valley K, denoted in Fig. S4a by $\Sigma_{12}(0)$, will therefore take the following form:

$$\Sigma_{12}(N) = \sum_{\gamma} \left[2\langle\gamma,\gamma_1|V|\gamma_1,\gamma\rangle - \langle\gamma,\gamma_1|V|\gamma,\gamma_1\rangle\right], \tag{S12}$$

where $\gamma = \{S = 1, k = 2\pi m/L, \sigma =\uparrow\}$ and $\gamma_1 = \{S_1 = 2, k_1 = 0, \sigma_1 =\uparrow\}$, and the first-subband wave vector m changes from $-m_F$ to m_F, that is, runs over all holes occupying the first subband symmetrically. The maximum index is related to the number of holes N in each valley by $N = 2m_F + 1$. The index m_1 of the second subband is zero, as we are adding the new hole to the bottom of the second subband. We note that there is a factor of two in front of the direct term. This accounts for the equal filling of both valleys and the fact that, as shown by Eq. (S10), the value of the direct Coulomb matrix element does not depend on the wave numbers of the two electrons. Let us denote a single direct term by U_{12}. This term is obtained from Eq. (S10) by setting $S = 1$ and $S_1 = 2$. As a result, the entire sum over direct elements evaluates to $2NU_{12}$. The sum over the exchange elements, on the other hand, is carried out only in one valley because of the spin selection rule. Utilizing the harmonic oscillator functions for our two subbands, we arrive at the selfenergy in the following form:

$$\Sigma_{12}(N) = 2NU_{12} - V_{12}^J(N),$$
$$V_{12}^J(N) = \frac{e^2}{4\pi\epsilon_0\epsilon_r L}\left[\frac{4\pi^2 l^2}{L^2}\right]\sum_{m_1=-m_F}^{m_F} \exp\left(\frac{2\pi^2 m_1^2 l^2}{L^2}\right)\int_{m_1}^{\infty} dv\sqrt{v^2 - m_1^2}\exp\left(-\frac{2\pi^2 l^2}{L^2}v^2\right).$$

The total direct contribution is proportional to the total number of holes. On the other hand, on closer inspection, we find that the sum $V_{12}^J(N)$ of exchange elements converges to a fixed value as m_F is increased (i.e., as the number N of holes in the lowest subband increases). This reflects the local nature of the exchange interaction.

3. *Interaction effects in intersubband gaps*

Evidently, in order to be able to calculate $\Sigma_{12}(N)$, we need to know the number of holes occupying our system at the gate voltage $V_{USG} = 10.3$ V. This number is not known a priori, but we can estimate it in the following way. Our point of departure is the fact that the addition of a hole into the channel happens only if the addition energy of that hole is equal to the Fermi energy of the leads. The first hole is added to the lowest subband at the gate voltage $V_{USG} = V_1 = 12$ V. In this case, since there are no other holes in the channel, the addition energy of that hole is simply equal to its single-particle energy. Therefore, the Fermi energy of the leads $E_F = E_{S=1}(k = 0, V_1)$. Now, we assume that E_F, being defined by the many-hole state in the leads, does not depend on the gate voltage V_{USG}. As the gate voltage is adjusted to $V_{USG} = V_2 = 10.3$ V, the hole is now added to the second subband, and so its addition energy must be equal to E_F at that point. Therefore, we can write

$$E_{S=2}(k = 0, V_2) + 2NU_{12} - V_{12}^J(N) = E_{S=1}(k = 0, V_1). \tag{S13}$$

We emphasize that the single-particle energy $E_{S=2}(k = 0)$ is extracted at the voltage $V_{USG} = V_2 = 10.3$ V, while $E_{S=1}(k = 0)$ is extracted at $V_{USG} = V_1 = 12$ V. In the absence of interactions, the above equation trivially simplifies to the equality of single-particle energies at the two voltages. This is easily understood: as the gate voltage V_{USG} is made less positive, the single-particle energies descend and align with E_F one by one, opening new conduction

channels. However, from our single-particle model we have $E_{S=2}(k=0,V_2) - E_{S=1}(k=0,V_1) = 0.3$ meV, that is, not zero. Therefore, the interaction component appearing on the left-hand side of Eq. (S13) must be negative to compensate for this mismatch in single-particle energies. In consequence, the total exchange $V^J_{12}(N)$ must be larger than the total direct term $2NU_{12}$. The numerical calculations of Coulomb elements require the length parameter, which for $V_{USG} = 10.3$ V is $l = 24.5$ nm. Assuming the dielectric constant $\varepsilon_r = 3.5$, we find $U_{12} = 3.45$ meV and $V^J_{12}(N)$ dependent nontrivially on N. For example, for $N = 11$, $V^J_{12}(N) = 4.837$ meV, for $N = 101$, $V^J_{12}(N) = 17.486$ meV, for $N = 201$, $V^J_{12}(N) = 22.057$ meV, and for $N = 301$, $V^J_{12}(N) = 24.756$ meV. Evidently, the total direct term $2NU_{12}$ is orders of magnitude larger than the total exchange term, and the negative interaction correction required in Eq. (S13) simply cannot be realized for any number N due to the excessively large value of U_{12}. If this were true in the experimental system, the addition to the second subband would never take place, since making V_{USG} even less positive (i.e., adding more holes into the lowest channel subband) only makes the alignment worse.

To address this discrepancy, we include additional screening of the direct interactions, while the exchange interactions remain screened only by the dielectric constant ε_r of the material. This screening originates from the nearby gates which maintain the holes inside the channel. Of course, we have to expect that the holes present in the system must not repel too strongly, otherwise we would not be able to see the essentially ballistic signatures of transport, characteristic for systems with near to zero repulsion. Therefore, we postulate that the direct elements have to be additionally scaled by a constant ε_S accounting for the screening. This choice, however, introduces another unknown into Eq. (S13) which requires a second equation to be able to find both N and ε_S.

The second equation can be formulated by looking again at the bottom-right hand panel of Fig. S4b. Up to now we have tracked the hole added to the bottom of the second subband, denoted by the hollow red arrow in the subband $S = 2$. However, the conduction channel through the lowest subband remains open: a hole can be added to the level just above the Fermi energy in the subband $S = 1$, as denoted by a hollow red arrow at the extreme right of the panel. Evidently, the addition energy of either hole must be the same:

$$E_{S=2}(k=0,V_2) + 2NU_{12} - V^J_{12}(N) = E_{S=1}(m=m_F+1,V_2) + 2NU_{11} - V^J_{11}(N). \tag{S14}$$

The left-hand side of this equation is the addition energy of the hole to the bottom of the second subband, while the right-hand side represents the addition energy of the hole to the edge of the filled first subband (one orbital above the Fermi momentum $k_F = 2\pi m_F/L$ in that subband). Here, the single-particle energy $E_{S=1}(m=m_F+1,V_2)$ corresponds to the hole added to the first available state in the subband $S = 1$ and is calculated at the gate voltage $V_{USG} = V_2 = 10.3$ V, just as the second-subband energy $E_{S=2}(k=0,V_2)$. The direct element in the lowest subband is $U_{11} = 3.95$ meV if the extra screening is not included. The exchange term at the edge of the lowest subband is

$$V^J_{11}(N) = \sum_{\gamma} \langle \gamma, \gamma_1 | V | \gamma, \gamma_1 \rangle, \tag{S15}$$

where $\gamma = \{S=1, k=2\pi m/L, \sigma=\uparrow\}$ and $\gamma_1 = \{S_1=1, k_1=2\pi(m_F+1)/L, \sigma_1=\uparrow\}$. The summation extends over the lowest-subband wave vectors from the negative Fermi momentum $-k_F$ to the positive Fermi momentum k_F. The new hole, referred to by the composite index γ_1, is placed on the first available single-particle state, i.e., one with the momentum $k_1 = 2\pi(m_F+1)/L$. The Fermi momentum is defined by the number N of holes per valley by $2m_F+1 = N$. We find that, similarly to the second subband, the exchange at the edge of the lowest subband depends on the number N of holes in the valley, but converges as N increases. For $\varepsilon_r = 3.5$ we find the following values: for $N = 11$, $V^J_{11}(N) = 12.331$ meV, for $N = 101$, $V^J_{11}(N) = 26.634$ meV, for $N = 201$, $V^J_{11}(N) = 31.225$ meV, and for $N = 301$, $V^J_{11}(N) = 33.924$ meV. Compared to the exchange $V^J_{12}(N)$ at the bottom of the second subband, the values of $V^J_{11}(N)$ are significantly larger, particularly for lower values of N.

We introduce explicitly the effective screening of the direct term and rewrite our two equations in the following form:

$$E_{S=1}(k=0,V_1) - \left[E_{S=2}(k=0,V_2) - V^J_{12}(N)\right] = 2N\frac{1}{\varepsilon_S}U_{12}, \tag{S16}$$

$$\left[E_{S=2}(k=0,V_2) - V^J_{12}(N)\right] - \left[E_{S=1}(k=k_F+1,V_2) - V^J_{11}(N)\right] = 2N\frac{1}{\varepsilon_S}U_{12}\left(\frac{U_{11}}{U_{12}} - 1\right). \tag{S17}$$

We divide these two equations sidewise and end up with one equation, which does not depend on ε_S and depends on the number of holes N only implicitly (through the exchange and single-particle energies). The direct terms enter only as the ratio $U_{11}/U_{12} = 1.145$. We find that our equation is solved for $2N = 130$ holes, i.e., 65 holes per valley. This number does not contradict our earlier description of symmetry-broken states, as it puts us firmly in the regime of the symmetric lowest-subband configuration, which becomes the ground state configuration of the lowest subband at $2N = 102$ holes. We insert the number of holes into Eq. (S16) and solve for the effective screening parameter, obtaining $\varepsilon_S = 31.35$. We find, within our model, that the direct term is strongly screened. This explains essentially ballistic transport spectra seen experimentally in spite of the fact that the channel contains hundreds of holes.

We map out the dependence of the effective screening constant ε_S on the overall strength of interactions, quantified by the dielectric constant ε_r. In the above analysis, we took $\varepsilon_r = 3.5$, and the effective screening ε_S acted on the direct interaction term on top of ε_r, while the exchange interaction was scaled by ε_r only. Using the same approach, we have computed ε_S and the critical number $2N$ of holes corresponding to the onset of the second subband for several model values of the dielectric constant ε_r. For $\varepsilon_r = 4.0$, we find $2N = 122$ and $\varepsilon_S = 30.39$. Further, for $\varepsilon_r = 5.5$, we find $2N = 106$ and $\varepsilon_S = 28.51$; for $\varepsilon_r = 7.0$, we find $2N = 96$ and $\varepsilon_S = 27.15$, and for $\varepsilon_r = 8.5$, we find $2N = 88$ and $\varepsilon_S = 25.94$. In general, as the interactions are made weaker, the critical number $2N$ of holes becomes smaller. In all cases, however, the onset of the transport involving the second subband occurs for a larger total number of holes than that corresponding to the phase transition to the symmetric configuration in the lowest subband. We find, therefore, no contradiction to the experimental data over a broad range of the system parameters. Furthermore, the effective screening parameter ε_S of the direct interactions appears to depend relatively weakly on the dielectric constant ε_r. Indeed, changing ε_r from 3.5 to 8.5, i.e., more than doubling it, leads to the decrease of ε_S only by about 17%. This is because the effective screening depends more on the ratios of different Coulomb elements rather than on their values, although, as is evident in Eq. (S16), this dependence involves also the single-particle energies and is therefore nontrivial. A more significant change of this parameter would be expected if we tuned the system so that the onsets of the first and second subbands correspond to altogether different gate voltages. Indeed, that would lead to different intersubband spacing, as well as different channel confining potentials, which would lead to a different length parameter l determining the Coulomb matrix elements. Therefore, the relative robustness of ε_S against tuning ε_r, but not gate voltages, suggests that this extra screening indeed originates from the gates, and would most likely be dependent on the geometry of the sample as well as the overall density of holes in the leads, all of these characteristics being gate-tunable.

4. Effective Single-Particle Model, the Anisotropic Massive Dirac Hamiltonian

As can be seen from the previous section, the interactions strongly modify the addition energies of the system. In particular, from Fig. S3a we see that the single-particle intersubband gaps are of order of 0.3 meV, the single-particle dispersion can span several meV, while the exchange corrections can be several times that value. To illustrate this, in Fig. S4c we show the energies of the two lowest channel subbands, S_1 (black) and S_2 (red) without interactions (dashed lines) and with interactions accounted for (solid lines). For the case without interactions, we simply plot $E_{S=1}(k)$ and $E_{S=2}(k)$ choosing the origin so that $E_{S=1}(0) = 0$. For the case with interactions, the system is filled with $2N = 130$ holes, in the symmetric configuration with $N = 65$ holes per valley. The blue dashed line denotes the Fermi energy corresponding to this occupation. We plot the quasiparticle energies:

$$
\begin{aligned}
E^{HF}_{S=1}(k) &= E_{S=1}(k) + \frac{N+N^*}{\varepsilon_S} U_{11} \\
&\quad - \sum_{\substack{k_1=-k_F \\ k_1 \neq k}}^{kF} \langle S=1, k_1, \uparrow; S=1, k, \uparrow |V| S=1, k_1, \uparrow; S=1, k, \uparrow \rangle, \qquad \text{(S18)}\\
E^{HF}_{S=2}(k) &= E_{S=2}(k) + \frac{2N}{\varepsilon_S} U_{12} \\
&\quad - \sum_{k_1=-k_F}^{kF} \langle S=1, k_1, \uparrow; S=2, k, \uparrow |V| S=1, k_1, \uparrow; S=2, k, \uparrow \rangle. \qquad \text{(S19)}
\end{aligned}
$$

The number N^* in the expression for $E^{HF}_{S=1}(k)$ equals $N-1$ when we are calculating the energy for $k \leq k_F$ (in the interior of the hole droplet), and N otherwise. This removes the selfinteraction effects in the direct interaction.

We see that the dispersion of the two lowest subbands is substantially changed by the interaction effects. Without interactions, the two lowest subbands are separated by a gap of order of 0.44 meV, which is approximately constant for all wave numbers k. Their quasi-parabolic dispersion can be characterized by the effective mass $m^* = 0.37\ m_0$, with m_0 being the free electron mass. On the other hand, the interactions open a large intersubband energy gap, of order of 22 meV, at $k = 0$. This gap strongly depends on the wave number: it decreases only slightly as a function of k in the interior of the droplet but decreases rapidly at its edge. The lower subband appears to be much more affected by the hole selfenergy than the upper one. Upon further increase of k, the two subbands approach each other. This characteristic behavior is caused mainly by the large difference in exchange energies for the lowest and second subbands. Moreover, the exchange being local, its renormalization of the quasiparticle energies decreases rapidly as we explore the quasiparticle energies further away from the Fermi energy. For large values of k, the two quasiparticle subbands are shifted from the ones for the noninteracting system by the direct term.

It is clear that this behavior of quasiparticle energies cannot be reproduced in detail by the simple massive Dirac Fermion model described by the Hamiltonian (S1). However, in reproducing the experimental results, we are only interested in the energy of the band edges, i.e., the bottom of each subband at $k = 0$. Moreover, the signatures of the addition steps appear when the holes begin to occupy the next unoccupied subband, as we discussed in the previous Section. Remarkably, we have found that a very good reproduction of the experimental results can be achieved by utilizing the anisotropic massive Dirac Fermion model presented in the main manuscript, with the Hamiltonian (S1) modified as follows:

$$\hat{H}_\tau^{(eff)} = \begin{bmatrix} \frac{\Delta}{2} + \alpha V(y) & \hbar v_F \tau \hat{k}_x - i\hbar v_F^{(eff)} \hat{k}_y \\ \hbar v_F \tau \hat{k}_x + i\hbar v_F^{(eff)} \hat{k}_y & -\frac{\Delta}{2} + \lambda + \alpha V(y) \end{bmatrix}. \tag{S20}$$

Here, $v_F^{(eff)}$ is the effective Fermi velocity in the y direction, that is, in the direction perpendicular to the channel. By tuning this parameter we account for the overall shift of the subband edge energy due to the direct and exchange contributions. We note that the dispersion along the channel is unchanged, which corresponds well to the approximately unchanged dispersion of the quasiparticle subbands close to the subband edge visible in Fig. S4b. We have achieved an excellent fit with the experimental data by choosing $\hbar v_F^{(eff)} = 0.15$ eV·nm, i.e., a value lower than the original one by a factor of 2.62. We assume, as previously, that the onset of the conductivity involving the first subband takes place at the gate voltage $V_{USG} = 12$ V which gives us the Fermi energy of the leads. The Fermi energy intersects the subsequent subbands at voltages corresponding very well to all the complete subband steps observed experimentally, as seen in Figs. 2(b-d) of the main text. Of course, without the inclusion of interactions, the single-particle model does not reproduce the symmetry breaking effects, and the subband onsets corresponds to the conductance steps of $2e^2/h$. While we match the model to the first conductance peak at $V_{USG} = 12$ V, the onset of the second subband is reproduced by our model to be between the single-height steps close to $V_{USG} = 10$ V. We stress that this effective model is set up only to reproduce the experimental peak positions, but does not reveal the microscopic nature of the shifts of subband edges relative to the bare single-particle model.

D. Symmetry Breaking for the Lowest Two Subbands

In this section, we expand the discussion of the symmetry breaking event, primarily focusing on the explanation of the terms found in equations 2 and 3 of the main text.

The first term in equations 2 and 3 of the main text is the total single-particle (kinetic) energy. Suppose that we have N holes in valley K. The states in that valley will be occupied up to a certain index m_M such that $2m_M + 1 = N$, and their total kinetic energy can be expressed as

$$T(N) = E_{S=1}(m = 0) + 2 \sum_{m=1}^{m_M} E_{S=1}(m) \tag{S21}$$

owing to the fact that the subband dispersion is symmetric. The energies $E_{S=1}(m)$ are calculated as appropriate eigenenergies of the Hamiltonian (S1).

Next, we compute the total direct interaction energy. The direct terms do not depend on the wave numbers of the subband states, only on their subband indices. Since we distribute all the holes on the first subband, the direct interaction of each pair is expressed by the same matrix element U_{11}. Therefore, irrespective of their distribution, the total direct repulsion energy of N holes will be

$$U(N) = \frac{N(N-1)}{2}U_{11}. \tag{S22}$$

Finally, we compute the total exchange interaction. For N holes in the valley K, this component of the total energy is expressed as

$$J(N) = \frac{1}{2}\sum_{m_1=-m_M}^{m_M}\sum_{\substack{m_2=-m_M\\ m_2\neq m_1}}^{m_M}\langle S=1, m_1, \uparrow; S=1, m_2, \uparrow |V| S=1, m_1, \uparrow; S=1, m_2, \uparrow\rangle. \tag{S23}$$

The exchange elements for the subbands $S = 1$ are obtained in a closed form. However, to obtain their numerical values, we need to establish the oscillator length l appropriate for our channel confinement. For the gate voltage $V_{USG} = 12$ V, we extract $l \approx 24$ nm. Furthermore, we assume that in the range of voltages $V_{USG} = 11$ to 12 V, the potential profile changes very little, and we can utilize the parameters and data generated for $V_{USG} = 12$ V throughout.

Now we formulate the total energies of the two configurations (equations 2 and 3 of the main text), $E^S_{HF}(N)$ for the symmetric one, and $E^A_{HF}(N)$ for the one with broken time-reversal symmetry. In the former case, we have $N/2$ holes in each valley, and

$$E^S_{HF}(N) = 2T(N/2) + U(N) - 2J(N/2). \tag{S24}$$

In the latter, all N holes are in one valley, and

$$E^{SB}_{HF}(N) = T(N) + U(N) - J(N). \tag{S25}$$

We find that each configuration has an identical direct energy $U(N)$, and we can disregard it. Therefore, the difference between these two total energies depends on the balance of the kinetic and exchange energies. We find that these two energies depend differently on the total number of holes N. The total kinetic energy increases approximately quadratically with N, since the dispersion is approximately parabolic, with the effective mass $m^* = 0.37\, m_0$ (with m_0 being the free electron mass). For a small N, $T(N)$ is in the μeV range, while for N large enough it attains the values in tens of meV. On the other hand, the exchange interaction of two holes on neighboring lowest-subband states is of order of 2.5 meV. However, the total exchange energy $J(N)$ increases with N quasi-linearly due to the short-range nature of the exchange. As a result, we expect strong exchange interaction effects at small N, while at larger N the difference between the energies $E^S_{HF}(N)$ and $E^{SB}_{HF}(N)$ will be mostly due to the difference in the total kinetic energy. This is what is seen and explained in the main text.

We have analyzed the stability of the spin and valley-polarized phase against the symmetrical one for the values of $\varepsilon_r = 4.0$, 5.5, 7.0, and 8.5, the latter being already larger than the theoretical estimates for the WSe_2 bulk dielectric constant. In all cases we find the symmetry-broken, valley-polarized phase to be the ground state for a small number of holes. The different values of ε_r only influence the critical number of holes for which the transition to the valley-symmetric configuration takes place. We find this number to be $N_C = 96$ for $\varepsilon_r = 4.0$, $N_C = 80$ for $\varepsilon_r = 5.5$, $N_C = 70$ for $\varepsilon_r = 7.0$, and $N_C = 64$ for $\varepsilon_r = 8.5$, for the first subband. Our HF analysis shows, therefore, that the symmetry-broken valley polarized phase is robustly stable over a broad range of system parameters.

Let us now turn to the splitting of the second subband. We will discuss two configurations analogous to those described by Eqs. (S24) and (S25), however the holes will be distributed both on the first ($S = 1$) and second ($S = 2$) subbands. When the signatures of the second subband appear in transport (at the gate voltage $V_{USG} \approx 10$ V), there are sufficiently many holes in the system for the occupation of the first subband to have transitioned to

the symmetric configuration. Moreover, we assume that the number of holes in the first subband is much larger than that in the second subband, which just begins to be populated at these gate voltages. The exchange energy experienced by the hole on the second-subband state $\gamma_2 = \{S = 2, k_2, \uparrow\}$ from all the holes on the lowest subband states $\gamma_1 = \{S = 1, k_1, \uparrow\}$ is expressed as

$$V_J^{2-1}(k_2) = \sum_{\gamma_2} \langle \gamma_1, \gamma_2 | V | \gamma_1, \gamma_2 \rangle. \tag{S26}$$

Now we account for the fact that the exchange interaction depends on the difference $k_1 - k_2$ of the wave numbers of single-particle states, and decreases rapidly with the increase of $|k_1 - k_2|$. As a result, for a very large first-subband occupation, the above energy does not depend on the index k_2. In other words, the holes on the first subband form a translationally invariant continuum, which corrects the energy of each second-subband state by the same amount. This approximation allows us to exclude the exchange term $V_J^{2-1}(k_2)$ (i.e., the interaction with the first subband) from the total HF energy of second-subband configurations. Thus, in comparing the energies of the symmetric and polarized second-subband configurations, we can use formulas analogous to Eqs. (S24) and S25), respectively, only with the kinetic and exchange energy specialized to the second subband. We perform our calculations by building the total kinetic energy with the dispersion calculated for the voltage $V_{USG} = 10$ V, and the total exchange energy with the length parameter $l = 24.5$ nm, appropriate for the region of interest.

As in the case of the lowest subband, we have analyzed the stability of the valley-polarized phase against the strength of interactions, quantified by the value of the dielectric constant ε_r. Again, adjusting this parameter influences the critical number N_C of electrons corresponding to the phase transition to the valley-symmetric configuration. We find this number to be $N_C = 38$ for $\varepsilon_r = 4.0$, $N_C = 32$ for $\varepsilon_r = 5.5$, $N_C = 28$ for $\varepsilon_r = 7.0$, and $N_C = 24$ for $\varepsilon_r = 8.5$. We again find that, in our HF analysis, the valley-polarized phase is robustly stable in our parameter space, however its stability region systematically shrinks as the interactions are made weaker.

III. NUMERICAL SOLUTIONS OF THE CHANNEL POTENTIAL

We simulate the potential landscape at the level of the monolayer WSe_2 by solving numerically the Laplace equation with boundary conditions corresponding to the chosen gate voltages, and assuming that the electric field approaches zero at a very large distance below the gate (von Neumann boundary conditions). Using the methodology described in details in Refs. (50, 51), and using the calculated dielectric constant of the hBN (3.5) under the gates, we generate the electrostatic potential $V(x, y)$ at the level of the WSe_2 flake. The cross-sections of $V(x, y)$ extracted along the red dashed line in Fig. S5a are plotted in Fig S5b for a fixed voltage of 4 V on the lower split gate and a varying voltage from 4 V to 12 V applied to the upper split gate. The complete potential landscape $V(x, y)$ is plotted when the upper split gate is set to 6.5 V (Fig. S5c) and 12 V (Fig. S5d) while the lower split gate is kept at 4 V.

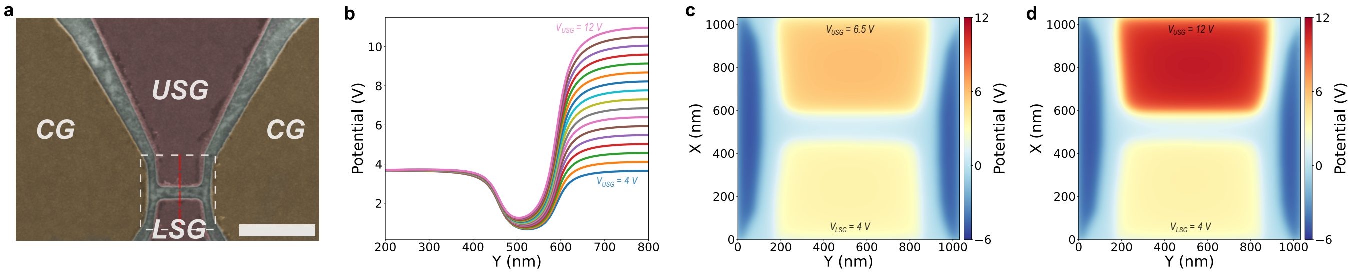

FIG. S5. **Potential Landscape. a)** False-color scanning electron micrograph of the top gate structure of the device. The scale bar corresponds to 1 μm. **b)** Profiles of the electrostatic potential at the level of the WSe_2 flake calculated along the red dashed line (a). The lower split gate is fixed at 4 V while the upper split gate varies from 4 V to 12 V at a 0.5 V interval. **c-d** Colormap of the electrostatic potential at the level of the WSe_2 flake of the white dashed area in (a). The lower split gate is fixed at 4 V while the upper split gate is fixed at 6.5 V (c) and 12 V (d).

IV. FINITE BIAS SPECTROSCOPY AND α-PARAMETER EXTRACTION

To obtain the lever arm of our device, a source-drain voltage, applied between contacts LC1 and RC2, is swept and the current traversing the device is measured. To eliminate the contribution from contact resistances, we monitor the voltage drop between contacts LC2 and RC3. The result is plotted in Fig. S6a as a function of the upper split gate where V_{Bias} is the voltage measured between LC2 and RC3. The conductance is obtained (Fig. S6b) by taking a numerical derivative with respect to the bias voltage. A second derivative, this time with respect to the split gate voltage, is performed to obtain the transconductance (Fig. S6c). From this plot, characteristic diamonds are visible (dashed black lines) where their slope corresponds to the lever arm:

$$\alpha = \frac{dV_{Bias}}{dV_{SG}} = 0.0012 \pm 0.0003$$

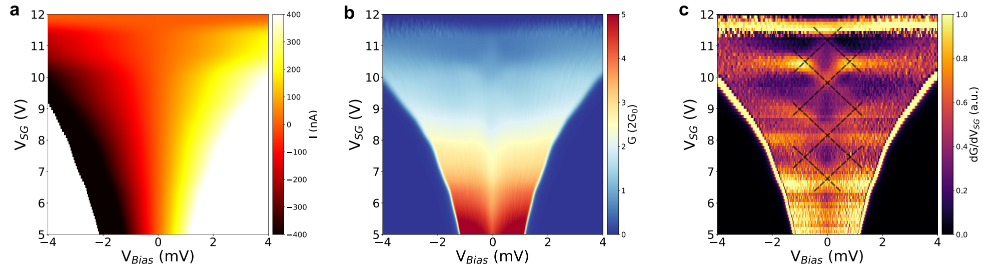

FIG. S6. **Finite Bias Spectroscopy of the 1D Channel.** **(a)** Current traversing the device as a function of the split gate voltage and the measured voltage across the channel. **(b)** Numerical derivatives with respect to the bias voltage and **(c)** with respect to the split gate voltage. Black dashed lines outline the characteristic diamonds.

V. EXCHANGE INTERACTION SCALING VS. CARRIER DENSITY

The Coulomb exchange matrix element is calculated as a function of the split gate-voltage and is shown to be well fitted by a quadratic function (Fig. S7a). From Fig. 3e of the main text, a clear linear relationship is found between the carrier density and the split-gate voltage. From these two tendencies, we find that the Coulomb exchange matrix element is related to the carrier density in the following form (dashed line in Fig. 3g of the main text):

$$J = -0.0011\left(\frac{n[10^{12}\,\mathrm{cm}^{-2}] + 0.66}{-0.11}\right)^2 + 0.038\left(\frac{n[10^{12}\,\mathrm{cm}^{-2}] + 0.66}{-0.11}\right) + 2.2$$

To observe this relation experimentally, we measure and plot the ratio w_T/w_1 as a function of the carrier density (Fig. 3g of the main text). The total width of the plateau w_T measured as a split gate voltage can be related to the energy spacing between the first and second subband of a single valley:

$$w_T = \alpha \Delta E_{2-1}$$

The energy spacing between the two subbands can be approximated as the energy spacing between two harmonic oscillator energy levels, which is related to the oscillator length l as:

$$w_T \propto \frac{\alpha \hbar}{m l^2}$$

The width w_1 directly probes the number of holes in the system at the transition between the SB-configuration and the S-configuration. We calculate this number of holes theoretically and find that it is linearly related to the inverse of the oscillator length (Fig. S7b), therefore:

$$w_1 \propto \frac{1}{l}$$

Furthermore, calculations show that the oscillator length is inversely proportional to the Coulomb exchange matrix element (Fig. S7c), therefore we expect the ratio w_T/w_1 to be proportional to J.

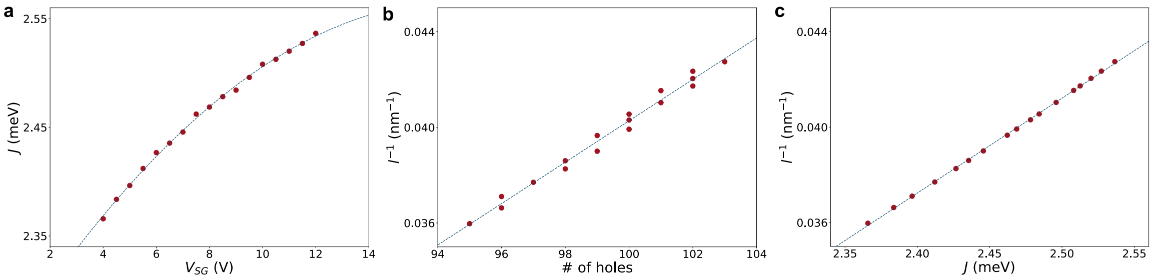

FIG. S7. **Scaling of the Coulomb exchange matrix element** Red data points represent calculated values for (a) the Coulomb exchange matrix element as a function of the upper split gate voltage, (b) the inverse of the oscillator length as a function of the number of holes at the transition, and (c) the inverse of the oscillator length as a function of the Coulomb exchange matrix element.

VI. TEMPERATURE DEPENDENCE

Conductance traces were recorded at various temperatures between 7 mK and 1 K (Fig. S8). Within this temperature range, the conductance plateaus appear unchanged.

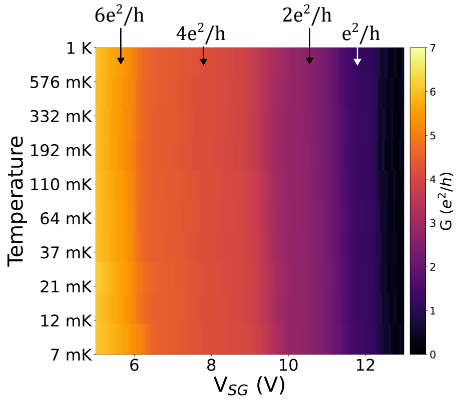

FIG. S8. **Temperature dependence** Temperature dependence, from base temperature to 1 K, of the quantized conductance plateaus, highlighting their robustness within this temperature range.

REFERENCES AND NOTES

1. S. Wu, J. S. Ross, G. B. Liu, G. Aivazian, A. Jones, Z. Fei, W. Zhu, D. Xiao, W. Yao, D. Cobden, X. Xu, Electrical tuning of valley magnetic moment through symmetry control in bilayer MoS_2. *Nat. Phys.* **9**, 149–153 (2013).

2. J. R. Schaibley, H. Yu, G. Clark, P. Rivera, J. S. Ross, K. L. Seyler, W. Yao, X. Xu, Valleytronics in 2D materials, *Nat. Rev. Mat.* **1**, 16055 (2016).

3. J. Lee, K. F.Mak, J. Shan, Electrical control of the valley Hall effect in bilayer MoS_2 transistors. *Nat. Nanotechnol.* **11**, 421–425 (2016).

4. S. A. Vitale, D. Nezich, J. O. Varghese, P. Kim, N. Gedik, P. Jarillo-Herrero, D. Xiao, M. S. R. A. Vitale, D. Nezich, J. O. Varghese, M. Rothschild, P. Kim, N. Gedik, P. Jarillo-Herrero, D. Xiao, Valleytronics: Opportunities, challenges, and paths forward, *Small* **14**, e1801483 (2018).

5. S. Lin, A. Carvalho, S. Yan, R. Li, S. Kim, A. Rodin, L. Carvalho, E. M. Chan, X. Wang, A. H. C. Neto, J. Yao, Accessing valley degree of freedom in bulk Tin(II) sulfide at room temperature. *Nat. Commun.* **9**, 1455 (2018).

6. M. S. Mrudul, M. Ivanov, A. Jiménez-Galán, G. Dixit, Light-induced valleytronics in pristine graphene. *Optica* **8**, 422 (2021).

7. A. Altıntaş, M. Bieniek, A. Dusko, M. Korkusinski, J. Pawłowski, P. Hawrylak, Spin-valley qubits in gated quantum dots in a single layer of transition metal dichalcogenides. *Phys. Rev. B* **104**, 195412 (2021).

8. N. Rana, G. Dixit, All-optical ultrafast valley switching in two-dimensional materials. *Phys. Rev. Appl.* **19**, 34056 (2023).

9. J. Pawłowski, J. E. Tiessen, R. Dax, J. Shi, Electrical manipulation of valley qubit and valley geometric phase in lateral monolayer heterostructures. *Phys. Rev. B* **109**, 045411 (2024).

10. D. Xiao, G.-B. Liu, W. Feng, X. Xu, W. Yao, Coupled spin and valley physics in monolayers of MoS_2 and other group-VI dichalcogenides. *Phys. Rev. Lett.* **108**, 196802 (2012).

11. A. Kormányos, G. Burkard, M. Gmitra, J. Fabian, V. Zólyomi, N. D. Drummond, V. Fal'ko, k · p theory for two dimensional transition metal dichalcogenide semiconductors. *2D Materials* **2**, 022001 (2015).

12. M. Van der Donck, F. M. Peeters, Interlayer excitons in transition metal dichalcogenide heterostructures. *Phys. Rev. B* **98**, 115104 (2018).

13. H. Zeng, J. Dai, W. Yao, D. Xiao, X. Cui, Valley polarization in MoS_2 monolayers by optical pumping. *Nat. Nanotechnol.* **7**, 490–493 (2012).

14. K. F. Mak, K. He, J. Shan, T. F. Heinz, Control of valley polarization in monolayer MoS_2 by optical helicity. *Nat. Nanotechnol.* **7**, 494–498 (2012).

15. T. Scrace, Y. Tsai, B. Barman, L. Schweidenback, A. Petrou, G. Kioseoglou, I. Ozfidan, M. Korkusinski, P. Hawrylak, Magnetoluminescence and valley polarized state of a two-dimensional electron gas in WS_2 monolayers, *Nat. Nanotechnol.* **10**, 603–607 (2015).

16. E.-M. Shih, Q. Shi, D. Rhodes, B. Kim, K. Watanabe, T. Taniguchi, K. Yang, J. Hone, C. R. Dean, Spin-selective magneto-conductivity in WSe_2. arXiv:2307.00446 [cond-mat.mes-hall] (2023).

17. B. A. Foutty, V. Calvera, Z. Han, C. R. Kometter, S. Liu, K. Watanabe, T. Taniguchi, J. C. Hone, S. A. Kivelson, B. E. Feldman, Anomalous Landau level gaps near magnetic transitions in monolayer WSe_2. *Phys. Rev. X* **14**, 031018 (2024).

18. R. Krishnan, S. Biswas, Y.-L. Hsueh, H. Ma, R. Rahman, B. Weber, Spin-valley locking for in-gap quantum dots in a MoS_2 transistor. *Nano Lett.* **23**, 6171–6177 (2023).

19. R. Pisoni, Y. Lee, H. Overweg, M. Eich, P. Simonet, K. Watanabe, T. Taniguchi, R. Gorbachev, T. Ihn, K. Ensslin, Gate-defined one-dimensional channel and broken symmetry states in MoS_2 van der Waals heterostructures. *Nano Lett.* **17**, 5008–5011 (2017).

20. K. Marinov, A. Avsar, K. Watanabe, T. Taniguchi, A. Kis, Resolving the spin splitting in the conduction band of monolayer MoS_2. *Nat. Commun.* **8**, 1938 (2017).

21. A. Epping, L. Banszerus, J. Güttinger, L. Krückeberg, K. Watanabe, T. Taniguchi, F. Hassler, B. Beschoten, C. Stampfer, Quantum transport through MoS_2 constrictions defined by photodoping. *J. Phys. Condens. Matter* **30**, 205001 (2018).

22. K. Sakanashi, P. Krüger, K. Watanabe, T. Taniguchi, G.-H. Kim, D. K. Ferry, J. P. Bird, N. Aoki, Signature of spin-resolved quantum point contact in p-type trilayer WSe_2 van der Waals heterostructure, *Nano Lett.* **21**, 7534–7541 (2021).

23. J. Boddison-Chouinard, A. Bogan, P. Barrios, J. Lapointe, K. Watanabe, T. Taniguchi, J. Pawłowski, D. Miravet, M. Bieniek, P. Hawrylak, A. Luican-Mayer, L. Gaudreau, Anomalous conductance quantization of a one-dimensional channel in monolayer WSe_2. *npj 2D Mat. Appl.* **7**, 50 (2023).

24. M. V. Gustafsson, M. Yankowitz, C. Forsythe, D. Rhodes, K. Watanabe, T. Taniguchi, J. Hone, X. Zhu, C. R. Dean, Ambipolar Landau levels and strong band-selective carrier interactions in monolayer WSe_2, *Nat. Mater.* **17**, 411–415 (2018).

25. Q. Shi, E.-M. Shih, M. V. Gustafsson, D. A. Rhodes, B. Kim, K. Watanabe, T. Taniguchi, Z. Papić, J. Hone, C. R. Dean, Odd- and even-denominator fractional quantum Hall states in monolayer WSe_2. *Nat. Nanotechnol.* **15**, 569–573 (2020).

26. J. Pack, Y. Guo, Z. Liu, B. S. Jessen, L. Holtzman, S. Liu, M. Cothrine, K. Watanabe, T. Taniguchi, D. G. Mandrus, K. Barmak, J. Hone, C. R. Dean, Charge-transfer contacts for the measurement of correlated states in high-mobility WSe_2. *Nat. Nanotechnol.* **19**, 948–954 (2024).

27. K. Kaasbjerg, K. S. Thygesen, K. W. Jacobsen, Phonon-limited mobility in *n*-type single-layer MoS_2 from first principles. *Phys. Rev. B* **85**, 115317 (2012).

28. H. C. P. Movva, A. Rai, S. Kang, K. Kim, B. Fallahazad, T. Taniguchi, K. Watanabe, E. Tutuc, S. K. Banerjee, High-mobility holes in dual-gated WSe_2 field-effect transistors. *ACS Nano* **9**, 10402–10410 (2015).

29. A. Y. Joe, K. Pistunova, K. Kaasbjerg, K. Wang, B. Kim, D. A. Rhodes, T. Taniguchi, K. Watanabe, J. Hone, T. Low, L. A. Jauregui, P. Kim, Transport study of charge-carrier scattering in monolayer WSe_2. *Phys. Rev. Lett.* **132**, 056303 (2024).

30. L. Szulakowska, M. Bieniek, P. Hawrylak, Electronic structure, magnetoexcitons and valley polarized electron gas in 2D crystals. *Solid State Electron.* **155**, 105–110 (2019).

31. M. Bieniek, M. Korkusiński, L. Szulakowska, P. Potasz, I. Ozfidan, P. Hawrylak, Band nesting, massive Dirac fermions, valley Lande and Zeeman effects in transition metal dichalcogenides: A tight-binding model. *Phys. Rev. B* **97**, 085153 (2018).

32. F. Rose, M. O. Goerbig, F. Piéchon, Spin- and valley-dependent magneto-optical properties of MoS_2. *Phys. Rev. B* **88**, 125438 (2013).

33. K. J. Thomas, J. T. Nicholls, M. Y. Simmons, M. Pepper, D. R. Mace, D. A. Ritchie, Possible spin polarization in a one-dimensional electron gas. *Phys. Rev. Lett.* **77**, 135 (1996).

34. C.-K. Wang, K.-F. Berggren, Local spin polarization in ballistic quantum point contacts. *Phys. Rev. B* **57**, 4552–4556 (1998).

35. S. M. Cronenwett, H. J. Lynch, D. Goldhaber-Gordon, L. P. Kouwenhoven, C. M. Marcus, K. Hirose, N. S. Wingreen, V. Umansky, Low-temperature fate of the 0.7 structure in a point contact: A Kondo-like correlated state in an open system. *Phys. Rev. Lett.* **88**, 226805 (2002).

36. P. Jaksch, I. Yakimenko, K.-F. Berggren, From quantum point contacts to quantum wires: Density-functional calcu lations with exchange and correlation effects. *Phys. Rev. B* **74**, 235320 (2006).

37. A. P. Micolich, What lurks below the last plateau: Experimental studies of the $0.7 \times 2e^2/h$ conductance anomaly in one-dimensional systems. *J. Phys. Condens. Matter* **23**, 443201 (2011).

38. F. Bauer, J. Heyder, E. Schubert, D. Borowsky, D. Taubert, B. Bruognolo, D. Schuh, W. Wegscheider, J. von Delft, S. Ludwig, Microscopic origin of the ‘0.7-anomaly’ in quantum point contacts. *Nature* **501**, 73–78 (2013).

39. A. Micolich, Double or nothing? *Nat. Phys.* **9**, 530–531 (2013).

40. D. H. Schimmel, B. Bruognolo, J. von Delft, Spin fluctuations in the 0.7 anomaly in quantum point contacts. *Phys. Rev. Lett.* **119**, 196401 (2017).

41. A. C. Graham, K. J. Thomas, M. Pepper, N. R. Cooper, M. Y. Simmons, D. A. Ritchie, Interaction effects at crossings of spin-polarized one-dimensional subbands. *Phys. Rev. Lett.* **91**, 136404 (2003).

42. A. Graham, K. Thomas, M. Pepper, M. Simmons, D. Ritchie, K.-F. Berggren, P. Jaksch, A. Debnarova, I. Yakimenko, 0.7 analogue structures and exchange interactions in quantum wires. *Solid State Commun.* **131**, 591–597 (2004).

43. A. Graham, K. Thomas, M. Pepper, M. Simmons, D. Ritchie, 0.7 structure in quantum wires observed at crossings of spin-polarised 1D subbands. *Physica E* **22**, 264–267 (2004).

44. H. C. P. Movva, B. Fallahazad, K. Kim, S. Larentis, T. Taniguchi, K. Watanabe, S. K. Banerjee, E. Tutuc, Density dependent quantum hall states and Zeeman splitting in monolayer and bilayer WSe_2, *Phys. Rev. Lett.* **118**, 247701 (2017).

45. J. Pawłowski, D. Moravet, M. Bieniek, M. Korkusinski, J. Boddison-Chouinard, L. Gaudreau, A. Luican-Mayer, P. Hawrylak, Interacting holes in a gated WSe_2 quantum channel: Valley correlations and zigzag Wigner crystal. *Phys. Rev. B* **110**, 125147 (2024).

46. Y. Hou, G. Wang, C. Ma, Z. Feng, Y. Chen, T. Filleter, Quantification of the dielectric constant of MoS_2 and WSe_2 nanosheets by electrostatic force microscopy. *Mater. Charact.* **193**, 112313 (2022).

47. A. Laturia, M. L. Van de Put, W. G. Vandenberghe, Dielectric properties of hexagonal boron nitride and transition metal dichalcogenides: From monolayer to bulk. *npj 2D Mat. Appl.* **2**, 6 (2018).

48. Y. Kang, D. Jeon, T. Kim, Direct observation of the thickness-dependent dielectric response of MoS_2 and WSe_2. *J. Phys. Chem. C* **124**, 18316–18320 (2020).

49. M. Bieniek, K. Sadecka, L. Szulakowska, P. Hawrylak, Theory of excitons in atomically thin semiconductors: Tight binding approach. *Nanomaterials* **12**, 1582 (2022).

50. J. Kyriakidis, M. Pioro-Ladriere, M. Ciorga, A. S. Sachrajda, P. Hawrylak, Voltage-tunable singlet-triplet transition in lateral quantum dots. *Phys. Rev. B* **66**, 035320 (2002).

51. J. H. Davies, I. A. Larkin, E. V. Sukhorukov, Modeling the patterned two-dimensional electron gas: Electrostatics. *J. Appl. Phys.* **77**, 4504 (1995).
